# Responses of soil fungal communities and functional guilds to ~160 years of natural revegetation in the Loess Plateau of China

**DOI:** 10.3389/fmicb.2022.967565

**Published:** 2022-09-02

**Authors:** Wen Yang, Longfei Diao, Yaqi Wang, Xitong Yang, Huan Zhang, Jinsong Wang, Yiqi Luo, Shuqing An, Xiaoli Cheng

**Affiliations:** ^1^College of Life Sciences, Shaanxi Normal University, Xi'an, China; ^2^Key Laboratory of Ecosystem Network Observation and Modeling, Institute of Geographic Sciences and Natural Resources Research, Chinese Academy of Sciences, Beijing, China; ^3^Department of Biological Sciences, Center for Ecosystem Science and Society, Northern Arizona University, Flagstaff, AZ, United States; ^4^School of Life Sciences, Nanjing University, Nanjing, China; ^5^School of Ecology and Environmental Sciences, Yunnan University, Kunming, China

**Keywords:** degraded ecosystems, fungal functional groups, FUNGuild, soil fungal richness and diversity, soil organic carbon and nitrogen, Loess Plateau

## Abstract

Natural revegetation has been widely confirmed to be an effective strategy for the restoration of degraded lands, particularly in terms of rehabilitating ecosystem productivity and soil nutrients. Yet the mechanisms of how natural revegetation influences the variabilities and drivers of soil residing fungal communities, and its downstream effects on ecosystem nutrient cycling are not well understood. For this study, we investigated changes in soil fungal communities along with ~160 years of natural revegetation in the Loess Plateau of China, employing Illumina MiSeq DNA sequencing analyses. Our results revealed that the soil fungal abundance was greatly enhanced during the later stages of revegetation. As revegetation progresses, soil fungal richness appeared first to rise and then decline at the climax *Quercus liaotungensis* forest stage. The fungal Shannon and Simpson diversity indexes were the lowest and highest at the climax forest stage among revegetation stages, respectively. Principal component analysis, Bray–Curtis similarity indices, and FUNGuild function prediction suggested that the composition, trophic modes, and functional groups for soil fungal communities gradually shifted along with natural revegetation. Specifically, the relative abundances of *Basidiomycota, Agaricomycetes*, *Eurotiomycetes*, and ectomycorrhizal fungi progressively increased, while that of *Ascomycota, Sordariomycetes*, *Dothideomycetes*, *Tremellomycetes*, saprotrophic, pathotrophic, arbuscular mycorrhizal fungi, and endophyte fungi gradually decreased along with natural revegetation, respectively. The most enriched members of *Basidiomycota* (e.g., *Agaricomycetes*, *Agaricales*, *Cortinariaceae*, *Cortinarius*, *Sebacinales*, *Sebacinaceae*, *Tricholomataceae*, *Tricholoma*, *Russulales,* and *Russulaceae*) were found at the climax forest stage. As important carbon (C) sources, the most enriched symbiotic fungi (particularly ectomycorrhizal fungi containing more recalcitrant compounds) can promote organic C and nitrogen (N) accumulation in soils of climax forest. However, the most abundant of saprotrophic fungi in the early stages of revegetation decreased soil organic C and N accumulation by expediting the decomposition of soil organic matter. Our results suggest that natural revegetation can effectively restore soil fungal abundance, and modify soil fungal diversity, community composition, trophic modes, and functional groups by altering plant properties (e.g., plant species richness, diversity, evenness, litter quantity and quality), quantity and quality of soil nutrient substrates, soil moisture and pH. These changes in soil fungal communities, particularly their trophic modes and functional groups along with natural revegetation, impact the accumulation and decomposition of soil C and N and potentially affect ecosystem C and N cycling in the Loess Plateau of China.

## Introduction

As the global deforestation rate has increased sharply over the last 100 years (Wall et al., 2020), innumerable natural forests were converted to agricultural lands. Further, agricultural land is increasingly being abandoned in recent decades, which has emerged as a global issue (Khorchani et al., 2022). Campbell et al. (2008) has estimated that the total area of abandoned agricultural lands on a global scale may be up to 4.72 million km^2^. Incessant deforestation and increasing abandoned agricultural lands provide a unique opportunity for the return of natural revegetation (Balami et al., 2021; Khorchani et al., 2022). Natural revegetation has been generally recognized as an effective strategy for the restoration of degraded lands and mitigation of climate change through the reversal of biodiversity loss (Niwaxu and Shackleton, 2019; Balami et al., 2021), reduction in soil erosion (Fu et al., 2017), rehabilitation of ecosystem productivity and soil nutrients (Sullivan et al., 2019), and other ecosystem services (Balami et al., 2021). As natural revegetation progresses, the composition, species richness, and diversity of plant communities are significantly altered (Crouzeilles et al., 2016; Krishna et al., 2020). These changes result in the modification of litter and root characteristics; soil physicochemical properties (Rosenzweig et al., 2016; Hu et al., 2022), soil carbon (C) and nitrogen (N) cycling (Rosenzweig et al., 2016; Sullivan et al., 2019; Khorchani et al., 2022); and soil microbial communities (Krishna et al., 2020; Balami et al., 2021; Nuñez et al., 2021; Cai et al., 2022).

Microbes are the dominant life forms in soil (Coban et al., 2022), which are widely proposed to be indicators of ecosystem health and the restoration levels of degraded ecosystems (Fierer et al., 2021), as well as ecosystem mediators that enhance the restoration of degraded lands (Coban et al., 2022). Soil fungi comprise an immense ecologically and functionally dynamic kingdom of eukaryotic organisms, with a global scale diversity that encompasses some 0.8–5.1 million species (Tedersoo et al., 2014; Khomich et al., 2017). Soil fungi play vital roles in maintaining ecosystem processes and functions (Tedersoo et al., 2014), particularly as relates to ecosystem nutrient cycling (Treseder and Lennon, 2015; Balami et al., 2021). For instance, soil saprotrophic fungi are key drivers that regulate nutrient cycling (e.g., C and N) as they possess enhanced capacities to decompose recalcitrant plant residues and soil organic matter (SOM), in contrast to soil bacteria (Bello et al., 2021; Hagenbo et al., 2022). Mycorrhizal hyphal biomass and the turnover of symbiotic fungi are the vital C sources that contribute to recalcitrant soil C sequestration, as ectomycorrhizal (ECM) fungi and arbuscular mycorrhizal fungi (AMF) contain relatively recalcitrant compounds (e.g., chitin and glomalin, respectively; Godbold et al., 2006; Hagenbo et al., 2022). Furthermore, ECM fungi form symbiotic relationships with the roots of woody plants, which drive nutrient cycling in forest ecosystems (Coban et al., 2022). AMF form symbioses with the roots of ~80% of vascular plant species and assist them with acquiring more soil nutrients, especially N and phosphorus (P; Coban et al., 2022; Yan et al., 2022), and enhancing their tolerance against various environmental pressures (Fall et al., 2021). As natural revegetation progresses, degraded lands experience drastic successional changes than naturally ecosystems (Guo et al., 2019). These rapid changes induce fundamental shifts in soil fungal communities and their functionalities that manipulate ecosystem nutrient cycling (Balami et al., 2021). Thus, an integrated assessment of the impacts of natural revegetation on soil fungal communities and their functional characteristics are critical to further elucidate the mechanisms that drive ecosystem nutrient cycling along with natural revegetation.

Soil fungal communities are significantly impacted by vegetation community composition, diversity, litter characteristics (Goodness et al., 2016; Hanif et al., 2019), and soil nutrient substrates (Goldmann et al., 2015; Yang et al., 2019; Balami et al., 2021), soil moisture (Brockett et al., 2012; Urbanová et al., 2015), pH (Geml et al., 2014; Maestre et al., 2015), and climate (Tedersoo et al., 2014). Climatic variables drive soil fungal community composition through their direct and indirect effects on plant and soil properties (Tedersoo et al., 2014). Different vegetation communities provide distinct litter and root exudations to advance nutrient uptake for soil fungi; thus, they strongly impact soil fungal communities (Wardle, 2006; Laforestlapointe et al., 2017; Mariotte et al., 2018). Plant attributes (e.g., plant species richness and diversity) have been confirmed as the essential drivers for soil fungal communities (Goodness et al., 2016). Generally, high plant species richness translates to the greater richness of soil fungal communities (Peay et al., 2013; LeBlanc et al., 2015; Goodness et al., 2016). Soil nutrient substrates are considered as overwhelming driving factors for soil fungal communities due to myriad soil fungi being saprophytic (Peay et al., 2013; Zimudzi et al., 2018). However, the responses of distinct fungal community taxa to the availability of nutrients differ (Thomson et al., 2015; Yang et al., 2019). For instance, *Ascomycota* prefer high-quality and simple substrates (e.g., low C:N; Thomson et al., 2015), and flourish in oligotrophic environments (Yang et al., 2019). Whereas, *Basidiomycota* are inclined to degrade low-quality and recalcitrant substrates (e.g., high C:N; Thomson et al., 2015; Llimós et al., 2021), and thrive in copiotrophic environments (Yang et al., 2019). Additionally, the availability of water and N in the soil can enhance the activities of soil fungal communities, while increasing their diversity and composition (Brockett et al., 2012; Poole et al., 2018). Soil pH is recognized as the most important influential predictors for soil fungal communities (Geml et al., 2014). Generally, soil fungal abundance and diversity decline under lower soil pH (Maestre et al., 2015). Identifying the factors which drive alterations in soil fungal abundance, diversity, community composition, trophic modes, and functional groups along with natural revegetation can be instrumental toward a comprehensive understanding of the driving mechanisms of natural revegetation on soil fungal communities.

The Loess Plateau of China spans 640,000 km^2^, and is one of the world’s most weathered, vulnerable, and degraded land regions due to its erodible soils and severe anthropogenic disturbances (Fu et al., 2017). To prevent the further deterioration of ecosystems, while reestablishing degraded lands, the Chinese Government initiated a series of conservation projects in the 1980’s (Fu et al., 2017). The “Grain for Green” program was the largest and well-known ecological engineering program in China, implemented in 1999 (Deng et al., 2014; Fu et al., 2017), which involved the conversion of croplands at inclines greater than 15° to grassland, shrubland, or forestlands through natural revegetation or afforestation (Deng et al., 2014). To date, this program has successfully converted more than 16,000 km^2^ of croplands to grasslands or forestlands in the Loess Plateau (Xu et al., 2018). Prior investigations manifested that natural revegetation strongly modified soil properties (Zhang et al., 2021), soil organic C and N stocks as well as stabilization (Zhu et al., 2021; Zhang et al., 2022). Other studies articulated responses of soil microbial, bacterial, and fungal communities to revegetation (Guo et al., 2019; Liu et al., 2019; Zhao et al., 2019; Xu et al., 2020). However, these investigations focused on active vegetation restoration (i.e., afforestation; Liu et al., 2019; Xu et al., 2020), and/or natural revegetation over the short-term (< 50 years; Guo et al., 2019; Zhao et al., 2019). The impacts of long-term (>150 years) natural revegetation on soil fungal abundance, diversity, and community composition, trophic modes, and functional groups remain unclear. Thus, it has not yet been clarified whether these factors drive ecosystem nutrient cycling (e.g., C and N). For this study, we hypothesized that long-term natural revegetation altered soil fungal abundance, diversity, community composition, trophic modes, and functional groups, which were likely driven by variations in properties of plant (e.g., plant species richness and diversity, litter and root characteristics), and the soil (e.g., quantity and quality of soil nutrient substrates). To test this hypothesis, we coupled the Illumina MiSeq sequencing of fungal internal transcribed spacers (ITS) and quantitative polymerase chain reactions (qPCR) to examine the soil fungal abundance, diversity, community composition, trophic modes, and functional groups along with ~160 years of natural revegetation from farmland to pioneer weed, herbage, shrub, early forest, and ultimately to climax forest in the Loess Plateau of China. Sixteen biotic and abiotic factors were determined to identify the key factors that drove the variations in soil fungal communities. The objectives of the present study were to appraise the following issues: (1) How long-term natural revegetation affects soil fungal abundance and diversity, and which revegetation stage exhibits the most enriched fungal abundance and diversity, respectively; (2) How the relative abundances of fungal community compositions are transformed under long-term natural revegetation; (3) What are the differences between soil fungal trophic modes as well as functional groups at distinct revegetation stages, and whether these differences impact the sequestration of soil C and N; and (4) Identifying pivotal factors which affect the variations in soil fungal communities under long-term natural revegetation.

## Materials and methods

### Study area

**Figure 1:**
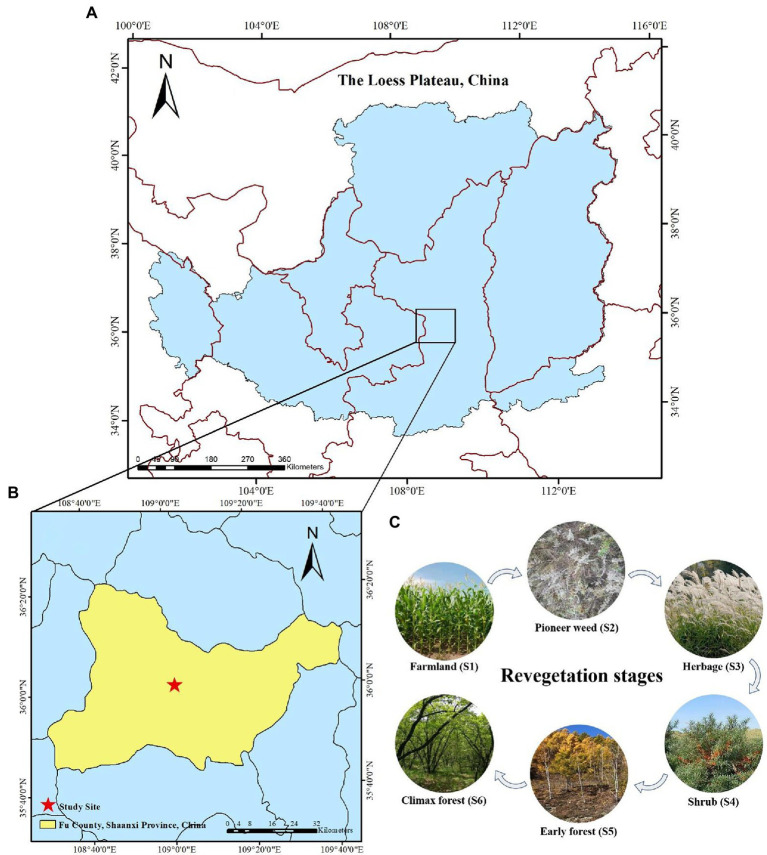
**(A)** Geographic location of the Loess Plateau, China, **(B)** study site in Fu County, Shanxi Province, China, **(C)** photographs of the study site at each natural revegetation stage.

This study was carried out in the Ziwuling Mountains (northwest face) located in Fu County of the Central Loess Plateau region (36° 00′ 14″–36° 01′ 09″ N and 109° 00′ 56″ – 109° 01′ 52″E) in Shaanxi Province, China (Figures 1A,B). This region is home to a temperate continental climate with average annual temperature and precipitation of 9°C and 576.7 mm, respectively, and average yearly sunlight duration of 2671.0 h (Yan et al., 2020). This area is characterized by typical hilly and gullied landscapes with altitudes that range from 1,157–1,396 m asl (above sea level; Yan et al., 2020). The soil of this area is the cinnamon type and categorized as Ustalfs according to the FAO Soil Taxonomy. The Ziwuling forest region is the most intact natural secondary forest region, spanning ~23,000 km^2^ in the Loess Plateau, which is due to undergo continued deforestation and intense soil erosion.

### Experimental design

During the Ming and Qing dynasties, natural vegetation nearly disappeared as the result of excessive deforestation and the transition of natural forests to farmlands. Since 1860, the abandonment of these lands occurred multiple times when the local population was displaced due to famine, war, and other calamities. Thus, over the last ~160 years vegetation has naturally recovered on these abandoned farmland, and various revegetation stages have been observed, ranging from pioneer weed, to herb, to shrub, to early, and climax forest communities (Zhong et al., 2018). Chen (1954) reported that *Populus davidiana* Dode was the dominant species in this region following ~100 years of natural revegetation. The ages of the pioneer weeds, herbs, and shrubs were determined through discussions with local elders and government departments regarding the land use history. We selected the six stages of natural revegetation as samples for this investigation: (1) farmland stage (control, 0 year means still farming, S1) where *Zea mays L*. was the primary rotation crop prior to revegetation; (2) pioneer weed stage (~15 years, S2) dominated by *Artemisia lavandulaefolia* DC. and *Stipa bungeana* Trin.; (3) herb stage (~30 years, S3) where *Miscanthus sacchariflorus* (Maximowicz) Hackel was the primary dominant species; (4) shrub stage (~50 years, S4), a *Hippophae rhamnoides* (Linn.) community was predominant; (5) early forest stage (~110 years, S5) dominated by *Populus davidiana* Dode; and (6) climax forest stage (~160 years, S6) dominated by *Quercus liaotungensis* Koidz. (Figure 1C). The geographical data and vegetation traits for each revegetation stage in this study are shown in Supplementary Table S1.

We conducted a field survey in October 2019, and four replicate plots were randomly established for each revegetation stage. To reduce the impacts of the localized site environments on the results, the distances between adjacent plots for each revegetation stage were not greater than 2 km, and the maximum relative elevation difference was <120 m. The sampling plots were determined by the community size: 20 m × 20 m for the early and climax forests, 5 m × 5 m for the shrub community, and 2 m × 2 m for the farmland, pioneer weed, and herb communities, respectively. For each revegetation phase, all plots were situated on similar slope gradients and aspects to ensure equivalent environments. Every plot for each restoration stage was at least 5 m away from each vegetation community boundary to avoid any edge effects.

### Soil sampling and soil properties analysis

Following the removal of litter layer, an S-shaped sampling technique was employed to randomly extract nine soil samples (Ø5 cm × 20 cm deep) from each plot at a 0–20 cm soil depth. Soil samples from each plot were thoroughly mixed to produce a single composite. All fresh soil samples were quickly sifted through a 2 mm sieve to eliminate visible debris and completely mixed, and were then placed in a dry-ice box and transferred to the laboratory. In the laboratory, soil samples were divided into four subsamples. The first soil subsample was immediately stored at −80°C for DNA extraction. The second soil subsample was used to determine soil moisture through oven drying at 105°C to a constant weight. The third soil subsample was stored at 4°C to quantify water-soluble organic carbon (WSOC). The fourth soil subsample was air-dried to measure the soil pH, soil organic carbon (SOC), total nitrogen (TN), soil organic nitrogen (SON), ammonium nitrogen (NH_4_^+^**-**N), nitrate nitrogen (NO_3_^−^**-**N), total phosphorus (TP), and SOC:SON. The soil pH value was quantified by a pH meter. The WSOC was measured according to the method described by Yang et al. (2016). Prior to determination of SOC and SON, the dried soil samples were treated at room temperature with 1 M HCl for 24 h to remove the total inorganic C, and then measured SOC and SON concentrations using a CN elemental analyzer (Vario PYRO cube elemental analyzer, Germany; Yang et al., 2019). The concentrations of TN, NH_4_^+^**-**N, NO_3_^−^**-**N, and TP were determined through the method described by Cai et al., (2022).

### Plant investigation and analysis

Along the soil sampling routes, three 1 × 1 m^2^ quadrats were stochastically selected from each plot for the early and climax forests, and shrub communities, respectively, to record the name, number, density, coverage, and frequency of each species. All the name, number, density, coverage, and frequency of each species were recorded in each of sampling plot (2 m × 2 m) for the farmland, pioneer weed, and herb communities, respectively. After recording, all litter in each quadrat were gathered, which were then cleaned thoroughly and oven dried at 65°C to a constant weight to measure litter biomass. A root sample core (Ø10 cm × 20 cm deep) from each quadrat of each plot were stochastically extracted with a root drill at soil depths of from 0 cm to 20 cm. Each root sample core was continuously rinsed with water using a 0.15 mm sieve, after which the remaining roots were gathered to determine the root biomass through oven drying at 65°C to a constant weight. Litter C:N ratio was calculated according to the C and N concentrations that were determined *via* a CN elemental analyzer (Vario PYRO cube elemental analyzer, Germany). The vegetation coverage (Coverage) was calculated using the proportion of understory vegetation projected to the ground per unit area (Xu et al., 2020). Plant species richness (PR) is the total number of species in each plot (Wang et al., 2019). Plant species diversity (PD) and evenness (PE) were calculated using the Shannon–Wiener Diversity Index and Pielou Evenness Index, respectively (Wang et al., 2019).

### DNA extraction and qPCR analysis

The total soil genomic DNA was extracted from a 0.5 g dry weight of frozen soil samples using the MoBio Power Soil DNA isolation kit in accordance with manufacturer’s protocols. The extracted soil DNA was divided into two parts, where the first DNA subsample was utilized for qPCR analysis, and the second was employed for Illumina MiSeq sequencing. The qPCR analysis specific for the soil fungal internal transcribed spacer (ITS) region was amplified using ITS1F primer (5S-CTTGGTCATTTAGAGGAAGTAA-3′) and ITS2R primer (5′-GCTGCGTTCTTCATCGATGC-3′) to quantify the soil fungal abundance (Gardes and Bruns, 1993). The total qPCR reaction contained 12.5 μl of SYBR Green qPCR Master Mix (2 ×), 2 μl of the DNA template, 0.5 μl each of 10 μM forward and reverse primers, and 9.5 μl of ddH_2_O, in a 25 μl final volume reaction. The ITS gene was amplified using an ABI 7500 real-time PCR system (Applied Biosystems, Foster City, CA, United States) with a program that provided an initial denaturing step at 95°C for 10 min., followed by 40 cycles at 95°C for 15 s, and finally 60°C for 1 min. All real-time PCR reactions were run in triplicate on the DNA extracted from each soil sample. The ITS gene copy number was calculated using the formula described by Yang et al. (2019).

### PCR amplification and Illumina MiSeq sequencing

The ITS1F primer (5S-CTTGGTCATTTAGAGGAAGTAA-3′) and ITS2R primer (5′-GCTGCGTTCTTCATCGATGC-3′) were designed to amplify the ITS region of soil fungal DNA. During the PCR process, the PCR components included 2 μl of 2.5 mM dNTPs, 0.4 μl of FastPfu Polymerase, 0.4 μl of 5 × FastPfu Buffer, 0.8 μl each of the forward and reverse primers (5 μM), 0.2 μl of bovine serum albumin, 10 ng of soil DNA, and sterile deionized H_2_O in a final 20 μl volume reaction. The PCR reaction was conducted in triplicate under the following conditions: 95°C for 3 min., with amplification that proceeded for 35 cycles at 94°C for 30 s, 55°C for 30 s, and 72°C for 45 s, followed by a final extension of 10 min. at 72°C using an ABI GeneAmp® 9,700 PCR System (Applied Biosystems, Foster City, United States). Following amplification, the PCR products were extracted from 2% agarose gels, purified using the AxyPrep DNA Gel Extraction Kit (Axygen Biosciences, Union City, CA, United States), and quantified using the QuantiFluor-ST blue fluorescence quantitative system (Promega, Madison, WI, USA). The purified amplicons were pooled in equimolar ratios and subjected to paired-end sequencing (2 × 250) with an Illumina MiSeq PE300 platform (Illumina Corporation, United States) from Shanghai Majorbio Bio-pharm Technology Co., Ltd., China.

### Bioinformatics

Sequences from the Illumina MiSeq platform processed using the Quantitative Insights Into Microbial Ecology (QIIME; v. 1.9.1) software package. The raw ITS gene sequences were demultiplexed, quality-filtered by using Trimmomatic v. 0.32, and then merged using FLASH (v. 1.2.11) under the criteria described by Yang et al. (2019). In total, 2,160,709 reads were acquired from the 24 soil samples using Illumina MiSeq sequencing. To obtain equivalent sequencing depth, the lowest number of reads (i.e., 60,563) in all subsets from each sample was arbitrarily chosen using the Mothur program (version 1.30.2), which ultimately produced 1,453,512 reads from 24 soil samples. The subsampled sequences were clustered using the UPARSE (v. 7.0.1090), and sequences with ≥97% similarity were assigned to the same operational taxonomic unit (OTU). To compare soil fungal alpha-diversity following the acquisition of 19,009 OTUs (in total) from the 24 soil samples, the Mothur program (v. 1.30.2; Schloss et al., 2009) was employed to calculate the OTU richness (total population of quantified OTUs), abundance-based coverage estimation (ACE), Chao’s species richness estimation (Chao1), as well as Shannon and Simpson diversity indexes (Shannon and Simpson, respectively). The fungal OTU taxonomic data was obtained using the Ribosomal Database Project (RDP) classifier (v. 11.5) by comparing the representative OTUs sequences with the UNITE Database (v. 8.0; Abarenkov et al., 2010). We employed linear discriminant analysis (LDA) coupled with effect size (LEfSe) to detect the effects of the treatments on soil fungal communities, at *p* < 0.05 and an LDA score of >4.0 (R software v. 3.2.2.). To infer potential functional traits, fungal OTUs were assigned to specific trophic modes, and were further subdivided into fungal functional guilds using the FUNGuild database (v.1.0; Nguyen et al., 2016; Yang et al., 2017). The confidence levels of “highly probable” or “probable” were selected in the FUNGuild fungal function prediction. Principal coordinate analysis (PCoA) of the OTUs was employed to assess the similarities in fungal community composition between samples using R software v. 3.2.2. Additionally, analysis of similarity (ANOSIM) tests was conducted with the vegan R package to determine the statistical differences in six revegetation stages using Bray-Curtis distances with 999 permutations, where the test statistic R, which measures the strength of the results, ranges from −1 to 1 (*R* = 1 signifies differences between groups, while *R* = 0 signifies that the groups are identical; Anderson and Walsh, 2013). A beta-diversity distance matrix was implemented to determine hierarchical clustering with the QIIME software package v. 1.9.1. Permutational multivariate analyses of variance (PERMANOVA, “adonis” in vegan R package) with 999 random permutations was used to test the effect of revegetation stages and environmental factors on soil fungal community variances. Finally, the complete dataset was deposited to the Sequence Read Archive database of the National Center for Biotechnology Information under the accession numbers of SRP388698.

### Statistical analysis

The statistical significance of the impacts of natural revegetation on plant and soil properties and soil fungal communities were analyzed with one-way analysis of variance using SPSS 24 software. Significant variations between the means of the groups were evaluated with Duncan’s test at *p* < 0.05. Redundancy analysis (RDA) was employed to investigate the relationships between soil fungal community compositions at the phylum-and class-level with plant and soil properties using CANOCO 4.5 software^1^. Pearson’s correlation analysis was implemented to determine the relationships between soil fungal abundance, alpha-diversity, and the relative abundances of the dominant trophic modes and guilds with the properties of plants and soil. Pearson’s correlation analysis between the relative abundances of fungal phyla and classes with plant and soil properties were shown as a heatmap (R software v. 3.2.2.).^2^

## Results

### Plant and soil properties

Plant species richness and diversity were highest in the early forest, and lowest in the farmland, respectively. Plant species evenness was highest in the shrub, and lowest in the farmland. The litter biomass, soil moisture, SOC, WSOC, and TN concentrations progressively increased along with natural revegetation. The root biomass was greatest in the climax forest, early forest, and farmland among revegetation stages. The litter C:N ratio in the climax forest was significantly higher than that of the early forest, shrub, and farmland. The soil pH was highest in the farmland, pioneer weed, and herbage stages, and lowest in the climax forest. The SON concentration and SOC:SON ratio in the climax and early forests were markedly higher than those for other revegetation stages. The soil NH_4_^+^**-**N concentration in the climax forest was considerably higher than that of the farmland, whereas the soil NO_3_^−^-N concentration was highest in the farmland and shrub stages among revegetation stages. The soil TP remained virtually unchanged along with natural revegetation (Supplementary Table S2).

### Abundance and alpha-diversity of soil fungal communities

The total soil fungal abundance was 1.72 × 10^7^ copies/g at the farmland stage, 1.79 × 10^7^ copies/g at the pioneer weed stage, 1.26 × 10^7^ copies/g at the herbage stage, 1.83 × 10^7^ copies/g at the shrub stage, 3.88 × 10^7^ copies/g at the early forest stage, and 1.17 × 10^8^ copies/g at the climax forest stage. The total soil fungal abundance was greatest in the climax forest among revegetation stages (Table 1).

**Table 1:** Number of sequences analyzed, observed soil (0–20 cm depth) fungal community abundance (fungal ITS gene copies per gram of soil), richness and diversity indices (mean ± SE, *n* = 4) during various revegetation stages in the Loess Plateau of China, obtained for clustering at 97% identity.

Revegetation stages	Gene of ITS 1–2 copies/g	Reads	OTU richness	Richness estimators		Diversity indices		Coverage (%)
				ACE	Chao1	Shannon	Simpson	
Farmland (S1)	1.722 × 10^7^ ± 1.242 × 10^6b^	60,563	755 ± 55^ab^	687 ± 33^b^	705 ± 30^b^	4.56 ± 0.07^a^	0.0284 ± 0.0045^b^	0.9984 ± 0.0001^a^
Pioneer weed (S2)	1.790 × 10^7^ ± 2.249 × 10^6b^	60,563	847 ± 22^a^	917 ± 27^a^	934 ± 29^a^	4.36 ± 0.25^a^	0.0680 ± 0.0275^ab^	0.9981 ± 0.0002^ab^
Herbage (S3)	1.257 × 10^7^ ± 2.527 × 10^6b^	60,563	768 ± 55^a^	837 ± 55^ab^	846 ± 53^ab^	4.64 ± 0.23^a^	0.0327 ± 0.0109^b^	0.9983 ± 0.0001^ab^
Shrub (S4)	1.832 × 10^7^ ± 2.420 × 10^6b^	60,563	860 ± 24^a^	920 ± 30^a^	938 ± 32^a^	4.95 ± 0.06^a^	0.0183 ± 0.0013^b^	0.9983 ± 0.0001^ab^
Early forest (S5)	3.878 × 10^7^ ± 1.209 × 10^7b^	60,563	902 ± 49^a^	993 ± 60^a^	1,000 ± 68^a^	4.52 ± 0.20^a^	0.0450 ± 0.0153^ab^	0.9977 ± 0.0003^b^
Climax forest (S6)	1.173 × 10^8^ ± 3.560 × 10^7a^	60,563	621 ± 63^b^	734 ± 75^b^	747 ± 85^b^	3.59 ± 0.17^b^	0.0951 ± 0.0274^a^	0.9977 ± 0.0003^b^
Source of variation	^**^	_	^*^	^**^	^**^	^**^	^*^	n.s.

Different superscript lower-case letters imply statistically significant differences at the *α* = 0.05 level among revegetation stages. Reads are the high-quality sequences following filtering and normalization. The richness estimators, diversity indices and coverage were calculated using the Mothur program. OTU richness: the total number of measured operational taxonomic units.

* *p* < 0.05;

** *p* < 0.01.

The fungal OTU richness was highest at the pioneer weed, herbage, shrub, and early forest stages, followed by the farmland stage, and lowest at the climax forest stage. Species richness indices (i.e., ACE and Chao1) at the pioneer weed, herbage, and early forest stages were markedly higher than those at the farmland and climax forest stages. The Shannon and Simpson diversity indexes of soil fungal communities were lowest and highest at the climax forest stage among revegetation stages, respectively. There was no significant difference in the Shannon diversity index of soil fungal communities between the pioneer weed, herbage, shrub, and early forest stages. The coverage of soil samples ranged from 99.77 to 99.84% across revegetation stages (Table 1).

### The composition of soil fungal communities

Across all soil samples, the fungal sequences belonged to 14 phyla, 46 classes, 123 orders, 307 families, 670 genera, and 1,117 species. The dominant phyla of the soil fungal communities across all revegetation stages were *Ascomycota* (48.31–70.75%), *Basidiomycota* (8.19–47.59%), *Mortierellomycota* (2.23–19.52%), and *Fungi_unclassified* (1.36–9.83%; Table 2). The relative abundance of *Ascomycota* at the farmland, pioneer weed, and shrub stages were markedly greater than that at the early and climax forest stages. The relative abundance of *Basidiomycota* was greatly increased at the climax and early forest stages compared to other revegetation stages. The relative abundance of *Mortierellomycota* was lowest at the climax forest and highest at the shrub stage. The relative abundance of *Olpidiomycota* was greatest at the shrub stage among revegetation stages (Table 2).

**Table 2:** Relative abundance (% of individual taxonomic groups) of the dominant fungal phyla (mean ± SE, *n* = 4) present in the soil (0–20 cm depth) microbial communities during various revegetation stages in the Loess Plateau of China.

Phylum	Revegetation stages						Source of variation
	Farmland (S1)	Pioneer weed (S2)	Herbage (S3)	Shrub (S4)	Early forest (S5)	Climax forest (S6)	
*Ascomycota*	70.75 ± 3.66^a^	66.69 ± 6.28^a^	59.70 ± 3.10^ab^	65.28 ± 3.35^a^	50.11 ± 5.52^b^	48.31 ± 5.66^b^	^*^
*Basidiomycota*	8.19 ± 1.49^c^	17.43 ± 5.05^c^	12.69 ± 1.36^c^	8.28 ± 1.23^c^	32.00 ± 6.07^b^	47.59 ± 6.19^a^	^**^
*Mortierellomycota*	15.89 ± 1.48^ab^	9.63 ± 1.14^c^	16.90 ± 2.53^ab^	19.52 ± 1.56^a^	12.52 ± 1.79^bc^	2.23 ± 0.29^d^	^***^
*Fungi_unclassified*	4.48 ± 0.67^b^	5.70 ± 1.73^b^	9.83 ± 0.99^a^	5.83 ± 0.76^b^	4.85 ± 0.87^b^	1.36 ± 0.31^c^	^**^
*Rozellomycota*	0.11 ± 0.07^a^	0.24 ± 0.17^a^	0.55 ± 0.48^a^	0.22 ± 0.12^a^	0.43 ± 0.29^a^	0.49 ± 0.31^a^	n.s.
*Olpidiomycota*	0.04 ± 0.01^b^	0.01 ± 0.01^b^	0.00 ± 0.00^b^	0.45 ± 0.32^a^	0.00 ± 0.01^b^	0.00 ± 0.00^b^	^*^
*Others*	0.55 ± 0.29^a^	0.30 ± 0.04^abc^	0.34 ± 0.03^abc^	0.41 ± 0.04^ab^	0.08 ± 0.02^bc^	0.01 ± 0.00^c^	^**^

n.s., not significant. Different superscript lower-case letters imply statistically significant differences at the *α* = 0.05 level among revegetation stages.

* *p* < 0.05;

** *p* < 0.01;

*** *p* < 0.001.

**Figure 2:**
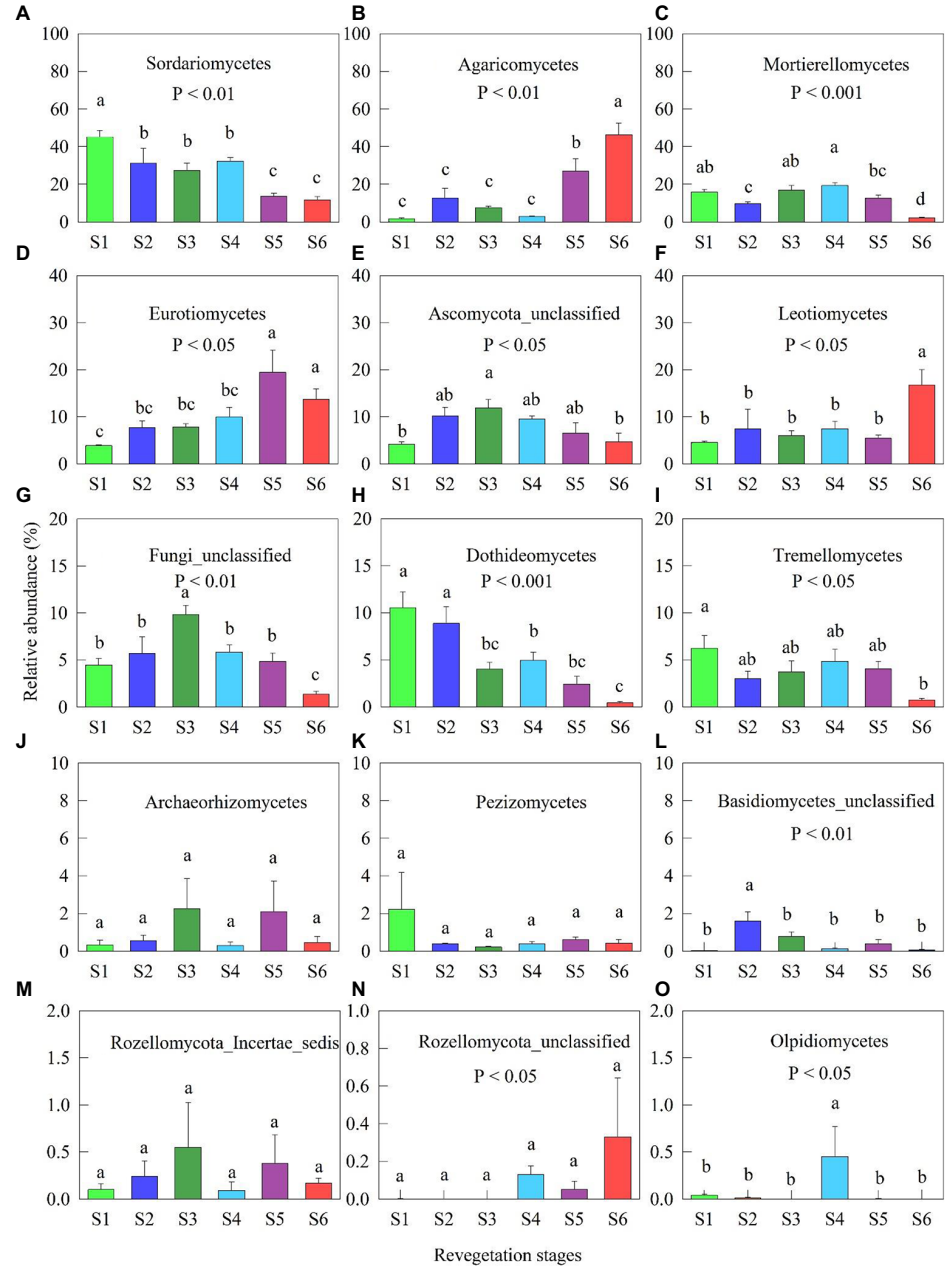
Relative abundance (% of individual taxonomic groups) of the dominant fungal classes (mean ± SE, *n* = 4) present in the microbial communities during various revegetation stages in the Loess Plateau of China. Different superscript lower-case letters imply statistically significant differences at the α = 0.05 level among revegetation stages. S1: Farmland stage (the control); S2: Pioneer weed stage; S3: Herb stage; S4: Shrub stage; S5: Early forest stage; and S6: Climax forest stage.

The high class levels of fungi belonged to *Sordariomycetes, Agaricomycetes, Mortierellomycetes, Eurotiomycetes, Ascomyota_unclassified, Leotiomycetes, Fungi_unclassified, Dothideomycetes,* and *Tremellomycetes* across all revegetation stages (Figure 2). The relative abundances of *Sordariomycetes* and *Dothideomycetes* gradually decreased along with the revegetation stages (Figures 2A,H). The relative abundances of *Agaricomycetes* and *Eurotiomycetes* at the early and climax forests stages were significantly enhanced compared with other revegetation stages (Figures 2B,D). The relative abundances of *Mortierellomycetes* and *Fungi_unclassified* were lowest at the climax forest stage among revegetation stages (Figures 2C,G). The relative abundance of *Leotiomycetes* at the climax forest stage was considerably higher than that at the other revegetation stages (Figure 2F). The relative abundance of *Tremellomycetes* was highest at the farmland, and lowest at the climax forest stage, respectively (Figure 2I).

At the family level, the relative abundances of *Mortierellaceae* and *Trichomeriaceae* were highest at the shrub stage and lowest at the climax forest stage. The relative abundances of *Chaetomiaceae, Piskurozymaceae, Didymellaceae, Lasiosphaeriaceae, Sporormiaceae, Trichosporonaceae, Stachybotryaceae,* and *Myrmecridiaceae* at the farmland stage were considerably higher than those at the other revegetation stages. The relative abundances of *Aspergillaceae, Inocybaceae, Trichocomaceae,* and *Thelephoraceae* were highest at the early forest stage. The relative abundance of *Nectriaceae* was highest at the farmland and shrub stages and lowest at the climax forest stage. The relative abundances of *Helotiaceae, Cortinariaceae, Herpotrichiellaceae, Sebacinaceae, Cordycipitaceae, Tricholomataceae, Russulaceae,* and *Sordariales_unclassified* were most enriched at the climax forest stage (Supplementary Table S3).

At the genus level, the relative abundance of *Mortierella* was highest at the shrub stage and lowest at the climax forest stage. The relative abundance of *Metarhizium* was highest at the pioneer weed stage and lowest at the early and climax forest stages. The relative abundances of *Penicillium*, *Inocybe*, *Talaromyces*, and *Clavulina* at the early forest stage were considerably greater than those at the other revegetation stages. The farmland possessed the highest relative abundances of *Chaetomiaceae_unclassified*, *Solicoccozyma*, *Fusarium*, *Preussia*, and *Chaetomium* among revegetation stages. The relative abundances of *Cortinarius*, *Helotiaceae_unclassified*, *Sebacina*, *Cladophialophora*, *Tricholoma*, *Beauveria*, *Russula*, and *Oidiodendron* were highest at the climax forest among revegetation stages (Table 3).

**Table 3:** Relative abundance (% of individual taxonomic groups) of the dominant fungal genera (mean ± SE, *n* = 4) present in the soil (0–20 cm depth) microbial communities during various revegetation stages in the Loess Plateau of China.

Genus	Revegetation stages						Source of variation
	Farmland (S1)	Pioneer weed (S2)	Herbage (S3)	Shrub (S4)	Early forest (S5)	Climax forest (S6)	
*Mortierella*	15.82 ± 1.50^ab^	9.55 ± 1.12^c^	16.75 ± 2.47^ab^	19.35 ± 1.56^a^	12.45 ± 1.76^bc^	2.22 ± 0.29^d^	^**^
*Ascomycota_unclassified*	4.13 ± 0.53^b^	10.14 ± 1.83^ab^	11.86 ± 1.80^a^	9.49 ± 0.69^ab^	6.52 ± 2.16^ab^	4.71 ± 1.81^b^	^*^
*Metarhizium*	4.66 ± 1.14^ab^	15.47 ± 7.58^a^	7.70 ± 4.11^ab^	6.62 ± 1.90^ab^	1.59 ± 0.13^b^	0.75 ± 0.55^b^	^**^
*Fungi_unclassified*	4.48 ± 0.67^b^	5.70 ± 1.73^b^	9.83 ± 0.99^a^	5.83 ± 0.76^b^	4.85 ± 0.87^b^	1.36 ± 0.31^c^	^**^
*Penicillium*	0.22 ± 0.14^c^	0.75 ± 0.40^c^	1.26 ± 0.18^c^	2.31 ± 0.76^bc^	8.41 ± 3.38^a^	6.48 ± 1.26^ab^	^**^
*Chaetomiaceae_unclassified*	12.70 ± 3.24^a^	1.17 ± 0.20^b^	1.38 ± 0.43^b^	1.85 ± 0.69^b^	0.46 ± 0.17^b^	0.14 ± 0.04^b^	^**^
*Cortinarius*	0.10 ± 0.05^b^	0.00 ± 0.00^b^	0.01 ± 0.01^b^	0.01 ± 0.01^b^	0.60 ± 0.27^b^	15.67 ± 9.25^a^	^**^
*Solicoccozyma*	4.90 ± 1.19^a^	1.69 ± 0.45^b^	1.50 ± 0.22^b^	2.37 ± 0.74^b^	2.31 ± 0.49^b^	0.31 ± 0.14^b^	^**^
*Inocybe*	0.03 ± 0.01^b^	0.00 ± 0.00^b^	0.00 ± 0.00^b^	0.01 ± 0.01^b^	10.55 ± 6.26^a^	3.36 ± 1.72^ab^	^**^
*Helotiaceae_unclassified*	0.07 ± 0.03^b^	0.00 ± 0.00^b^	0.00 ± 0.00^b^	0.00 ± 0.00^b^	0.01 ± 0.01^b^	13.14 ± 3.56^a^	^**^
*Fusarium*	3.93 ± 1.01^a^	1.55 ± 0.21^bc^	2.28 ± 0.25^b^	1.77 ± 0.12^bc^	0.81 ± 0.13^cd^	0.07 ± 0.01^d^	^**^
*Sebacina*	0.04 ± 0.01^b^	0.00 ± 0.00^b^	0.00 ± 0.00^b^	0.01 ± 0.01^b^	2.55 ± 1.03^b^	7.71 ± 2.79^a^	^**^
*Cladophialophora*	0.18 ± 0.04^c^	1.61 ± 0.43^b^	0.79 ± 0.25^bc^	0.65 ± 0.31^bc^	1.71 ± 0.27^b^	4.20 ± 0.75^a^	^**^
*Tricholoma*	0.04 ± 0.02^b^	0.00 ± 0.00^b^	0.00 ± 0.00^b^	0.00 ± 0.00^b^	0.06 ± 0.03^b^	7.39 ± 4.75^a^	^*^
*Talaromyces*	0.23 ± 0.07^b^	0.73 ± 0.63^b^	0.07 ± 0.02^b^	0.33 ± 0.11^b^	3.88 ± 1.32^a^	1.23 ± 0.40^b^	^*^
*Beauveria*	0.10 ± 0.04^b^	0.57 ± 0.24^b^	0.18 ± 0.05^b^	0.66 ± 0.30^b^	0.05 ± 0.02^b^	3.04 ± 0.98^a^	^**^
*Russula*	0.03 ± 0.01^b^	0.00 ± 0.00^b^	0.00 ± 0.00^b^	0.00 ± 0.00^b^	0.00 ± 0.00^b^	5.46 ± 1.80^a^	^**^
*Preussia*	2.42 ± 0.88^a^	0.28 ± 0.14^b^	0.27 ± 0.09^b^	0.20 ± 0.04^b^	0.02 ± 0.01^b^	0.01 ± 0.01^b^	^**^
*Oidiodendron*	0.02 ± 0.01^b^	0.06 ± 0.05^b^	0.04 ± 0.04^b^	0.04 ± 0.02^b^	0.93 ± 0.76^ab^	2.17 ± 0.85^a^	^*^
*Clavulina*	0.01 ± 0.01^b^	0.00 ± 0.00^b^	0.00 ± 0.00^b^	0.00 ± 0.00^b^	1.95 ± 1.02^a^	1.28 ± 0.72^ab^	^*^
*Chaetomium*	2.34 ± 0.28^a^	0.14 ± 0.02^b^	0.29 ± 0.10^b^	0.30 ± 0.06^b^	0.06 ± 0.03^b^	0.00 ± 0.00^b^	^**^
*Tomentella*	0.01 ± 0.00^b^	0.00 ± 0.00^b^	0.00 ± 0.00^b^	0.03 ± 0.01^b^	1.40 ± 0.28^a^	0.97 ± 0.25^a^	^**^

n.s., not significant. Different superscript lower-case letters imply statistically significant differences at the *α* = 0.05 level among revegetation stages.

* *p* < 0.05;

** *p* < 0.01.

### Statistically different soil fungal groups

**Figure 3:**
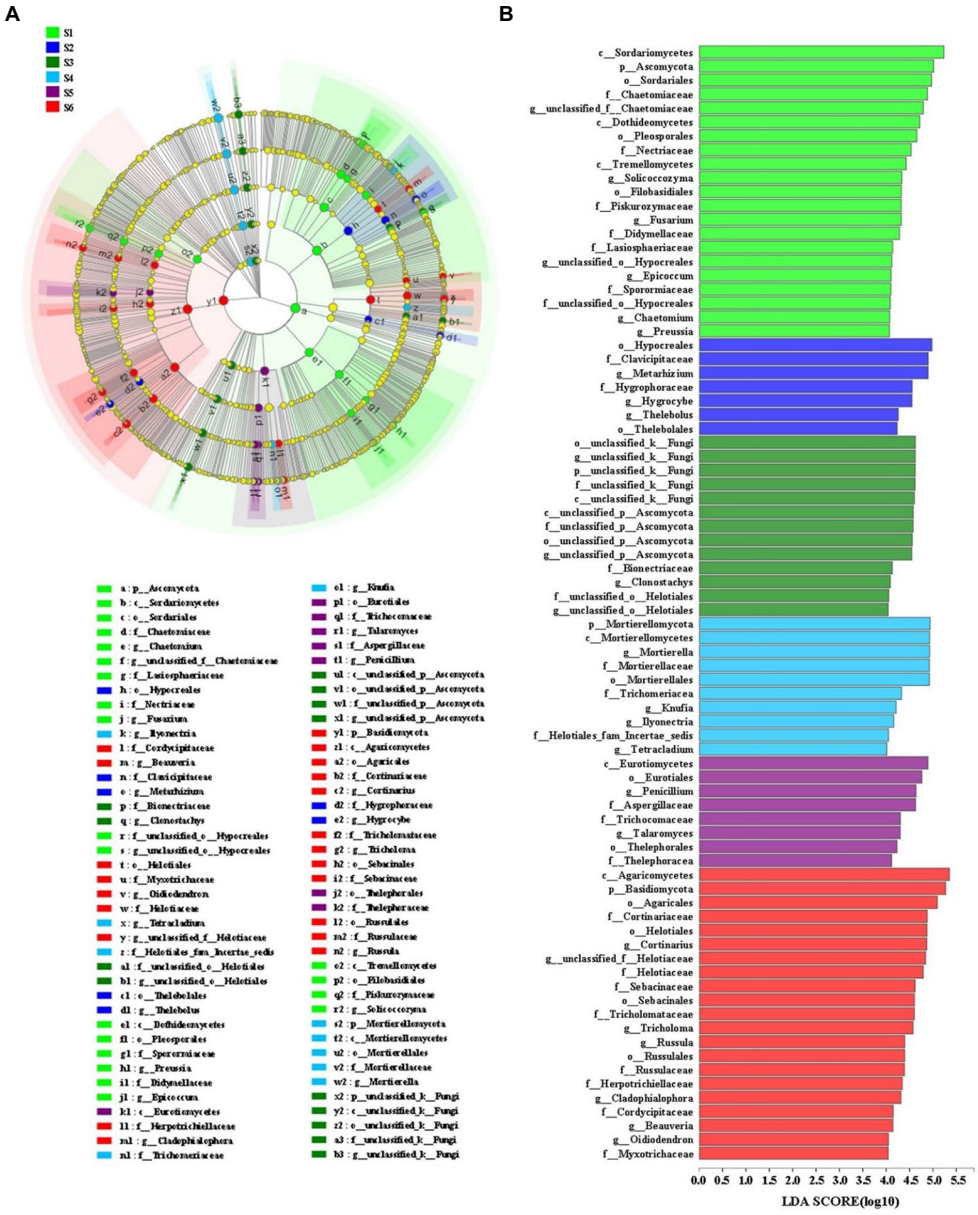
**(A)** Cladogram indicating the phylogenetic distribution of microbial lineages associated with six different restoration stages on the Loess Plateau, China; **(B)** lineages with LDA values of 4.0 or higher determined by LEfSe are displayed. Differences are represented by the color of the most abundant microbial groups (in Figure 3, emerald green indicates farmland stage (S1), dark blue indicates pioneer weed stage (S2), dark green indicates herbage stage (S3), sky blue indicates shrub stage (S4), dark purple indicates early forest stage (S5), red indicates climax forest stage (S6), and yellow indicates non-significant). Each circle’s diameter is proportional to the abundance of the taxon. Circles represent phylogenetic levels from phylum to genus, from the inside out.

LEfSe analyses was combined with LDA values to identify statistical significance of differentially abundant taxa for soil fungal communities among the various revegetation stages (Figure 3). Soil fungal communities in the climax forest were mainly enriched with *Basidiomycota, Agaricomycetes* (from class to order; within *Basidiomycota*), *Cortinariaceae* (from family to genus; within *Agaricomycetes*), *Helotiales* (from order to genus; *Ascomycota*), *Sebacinales* (from order to family; within *Agaricomycetes*), *Tricholomataceae* (from family to genus; within *Agaricomycetes*), *Russulales* (from order to genus; within *Agaricomycetes*). Fungal taxa that primarily clustered in early forest included *Eurotiomycetes* (from class to order; within *Ascomycota*), *Penicillium* and *Talaromyces* (genus within *Eurotiomycetes*), *Aspergillaceae* and *Trichocomaceae* (families within *Eurotiomycetes*), and *Thelephorales* (from order to family; within *Agaricomycetes*). Soil fungal taxa enriched at the shrub stage included *Mortierellomycota* (from phylum to genus), *Trichomeriacea* (a family within *Eurotiomycetes*), *Knufia* (a genus within *Eurotiomycetes*), *Ilyonectria* (a genus within *Sordariomycetes*), *Helotiales_fam_Incertae_sedis* (a family within *Leotiomycetes*), and *Tetracladium* (a genus within *Leotiomycetes*). The enriched fungal taxa in the herbage soil were *unclassified_k_Fungi* (from phylum to genus), *unclassified_p_Ascomycota* (from class to genus), *Bionectriaceae* (a family within *Sordariomycetes*), *clonostachys* (a genus within *Sordariomycetes*), and *unclassified_o_Helotiales* (from family to genus; within *Leotiomycetes*). Soil fungal communities at the pioneer weed stage were enriched with *Hypocreales* (an order within *Sordariomycetes*), *Clavicipitaceae* (a family within *Sordariomycetes*), *Metarhizium* (a genus within *Sordariomycetes*), *Hygrophoraceae* (from family to genus; within *Agaricomycetes*), and *Thelebolales* (order and genus; within *Leotiomycetes*). The enriching fungal groups in the farmland soil included *Ascomycota, Sordariomycetes* (from class to order; within *Ascomycota*), *Dothideomycetes* (within *Ascomycota*), *Tremellomycetes* (within *Basidiomycota*), *Chaetomiaceae* (from family to genus), *Pleosporales* (an order within *Dothideomycetes*), *Nectriaceae* (a family within *Sordariomycetes*), *Solicoccozyma* (within *Tremellomycetes*), *Filobasidiales* (within *Tremellomycetes*), *Piskurozymaceae* (a family within *Tremellomycetes*), *Didymellaceae* and *Sporormiaceae* (families within *Dothideomycetes*), *Fusarium* (a genus within *Sordariomycetes*), *Lasiosphaeriaceae* (a family within *Sordariomycetes*), *unclassified_o_Hypocreales* (from family to genus; Figure 3).

### Trophic modes and functional guilds of fungi communities

**Figure 4:**
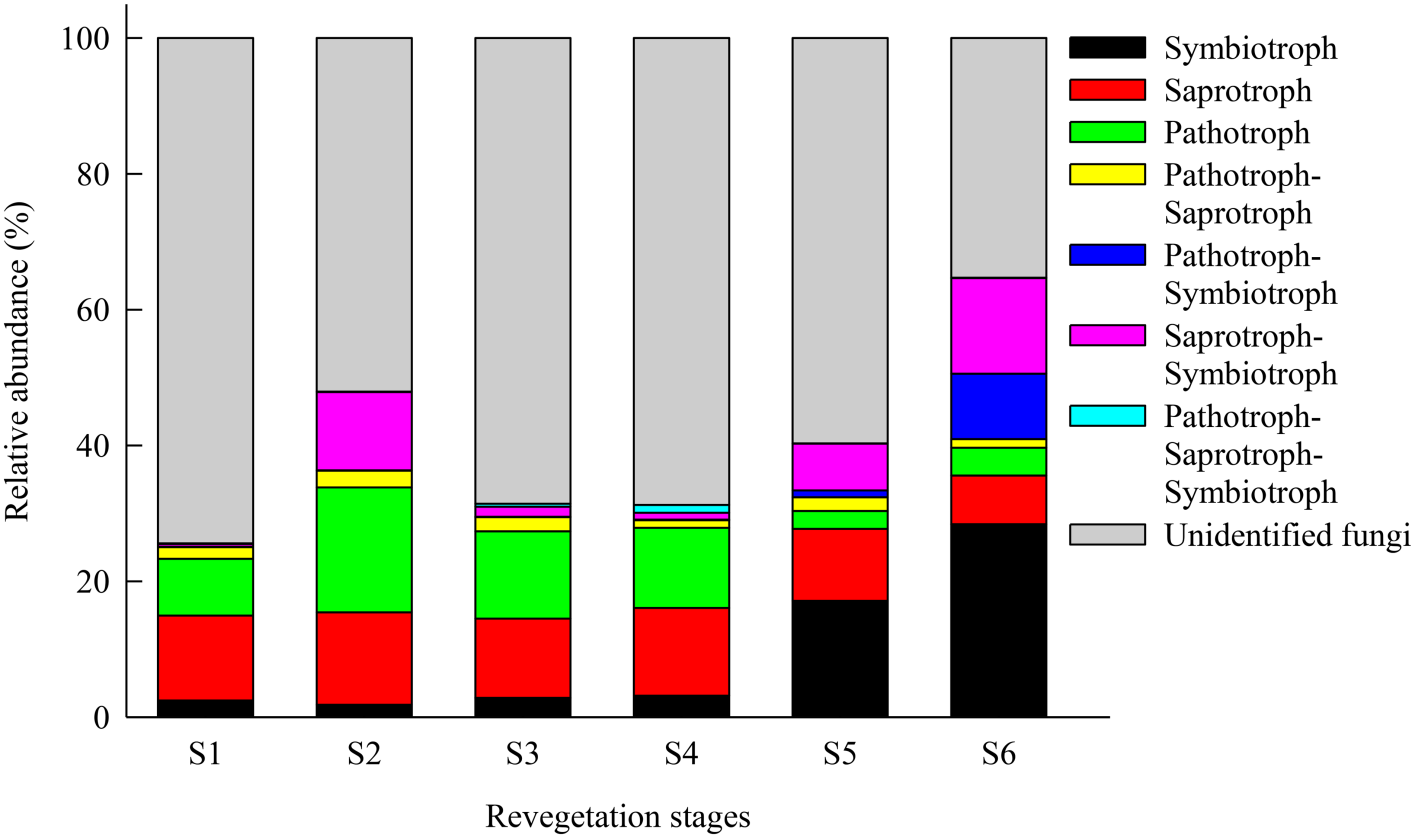
Relative abundance (%, sequences) of the corresponding fungal trophic modes (mean ± SE, *n* = 4) in the soil (0–20 cm depth) microbial communities during various revegetation stages in the Loess Plateau of China, inferred by FUNGuild. Different superscript lower-case letters imply statistically significant differences at the α = 0.05 level among revegetation stages. Figure 2 for abbreviations.

Soil fungal trophic modes and functional guilds at the OTUs level were inferred using FUNGuild. In this study, a total of 26, 48, 31, 31, 40, and 65% OTUs from the farmland, pioneer weed, herbage, shrub, early, and climax forest stages, respectively, were assigned to 7 trophic modes, and the remainder were undefined fungi. The relative abundances of symbiotrophs at the climax forest (28.42%) and early forest (17.12%) stages were markedly higher than those at the shrub (3.16%), herbage (2.82%), pioneer weed (1.82%), and farmland (2.43%) stages. The relative abundance of saprotrophs was highest at the pioneer weed stage (13.61%), and lowest at the climax forest stage (7.15%) among revegetation stages. The relative abundance of pathotrophs was highest at the pioneer weed stage (18.40%), followed by herbage (12.85%), shrub (11.80%), farmland (8.35%) stages, and lowest at the early and climax forest stages (2.64 and 4.10%, respectively) stages (Figure 4).

In terms of fungal functionality, 12 fungal functional guilds were identified from symbiotroph, saprotroph, and pathotroph trophic groups. The relative abundance of ectomycorrhizal (ECM) fungi at the climax forest stage was considerably higher than that at the other revegetation stages. The relative abundances of AMF and dung saprotroph-soil saprotroph fungi were most enriched at the shrub stage, and lowest at the climax forest stage. The relative abundance of endophytes was highest at the farmland stage and lowest at the climax forest stage. The relative abundance of animal pathogens was highest at the pioneer weed stage, and lowest at the early and climax forest stages. The relative abundance of fungal parasites at the pioneer weed and herbage stages was considerably higher than that at the climax forest stage (Table 4).

**Table 4:** Relative abundance (%, sequences; mean ± SE, *n* = 4) of assigned fungal functional guilds in symbiotroph, saprotroph and pathogen in the soil (0–20 cm depth) microbial communities during various revegetation stages in the Loess Plateau of China, inferred by FUNGuild.

Guild	Revegetation stages						Source of variation
	Farmland (S1)	Pioneer weed (S2)	Herbage (S2)	Shrub (S4)	Early forest (S5)	Climax forest (S6)	
ECM	0.23 ± 0.06^b^	0.33 ± 0.12^b^	1.05 ± 0.90^b^	1.07 ± 0.43^b^	16.67 ± 6.51^ab^	28.37 ± 9.01^a^	^**^
AMF	0.16 ± 0.04^bc^	0.13 ± 0.02^cd^	0.25 ± 0.03^ab^	0.28 ± 0.06^a^	0.05 ± 0.01d^e^	0.00 ± 0.00^e^	^***^
Endophyte	0.91 ± 0.55^a^	0.25 ± 0.07^ab^	0.17 ± 0.05^ab^	0.62 ± 0.22^ab^	0.12 ± 0.03^ab^	0.03 ± 0.01^b^	^**^
Dung saprotroph	1.35 ± 0.10^a^	0.08 ± 0.05^b^	0.10 ± 0.05^b^	0.05 ± 0.03^b^	0.01 ± 0.01^b^	0.00 ± 0.00^b^	^**^
Dung saprotroph-plant saprotroph	2.63 ± 0.87^a^	0.75 ± 0.19^b^	0.34 ± 0.11^b^	0.42 ± 0.08^b^	0.07 ± 0.02^b^	0.02 ± 0.01^b^	^**^
Dung saprotroph-soil saprotroph	0.28 ± 0.07^ab^	0.10 ± 0.03^bc^	0.12 ± 0.03^bc^	0.41 ± 0.19^a^	0.03 ± 0.01^bc^	0.00 ± 0.00^c^	^**^
Undefined saprotroph	6.84 ± 0.72^a^	10.79 ± 3.68^a^	7.44 ± 1.41^a^	10.09 ± 1.50^a^	7.87 ± 1.14^a^	6.28 ± 0.87^a^	n.s.
Undefined saprotroph-wood saprotroph	0.48 ± 0.12^a^	0.80 ± 0.50^a^	1.09 ± 0.74^a^	0.91 ± 0.36^a^	0.24 ± 0.11^a^	0.05 ± 0.03^a^	n.s.
Wood saprotroph	0.33 ± 0.20^a^	0.28 ± 0.11^a^	0.19 ± 0.02^a^	0.41 ± 0.15^a^	0.23 ± 0.08^a^	0.30 ± 0.15^a^	n.s.
Animal pathogen	4.82 ± 1.21^ab^	16.06 ± 7.50^a^	7.89 ± 4.06^ab^	7.38 ± 1.91^ab^	1.68 ± 0.15^b^	3.80 ± 1.48^b^	^*^
Plant pathogen	3.05 ± 0.07^a^	1.65 ± 0.67^a^	4.49 ± 2.97^a^	3.78 ± 0.90^a^	0.76 ± 0.16^a^	0.21 ± 0.09^a^	^**^
Fungal parasite	0.20 ± 0.06^ab^	0.41 ± 0.19^a^	0.38 ± 0.08^a^	0.36 ± 0.13^ab^	0.11 ± 0.03^ab^	0.04 ± 0.02^b^	^*^

n.s., not significant. ECM, ectomycorrhizal fungi; AMF, arbuscular mycorrhizal fungi. Different superscript lower-case letters imply statistically significant differences at the *α* = 0.05 level among revegetation stages.

* *p* < 0.05;

** *p* < 0.01;

*** *p* < 0.001.

### Beta-diversity of soil fungal communities

**Figure 5:**
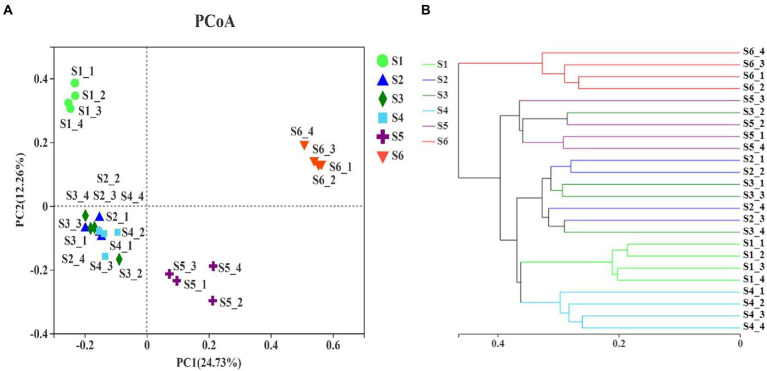
**(A)** Principal coordinate analysis and **(B)** clustering of samples. Bray–Curtis similarity index was calculated using OTU reads, and hierarchical clustering was calculated using the β-diversity distance matrix with QIIME. Figure 2 for abbreviations.

PCoA and Bray–Curtis similarity indices were employed to analyze the beta-diversity of soil fungal communities based on the OTU levels, and to identify differences between fungal communities across different revegetation stages (Figure 5). Both analyses revealed that climax forest soil was closely clustered; thus, it was distinct from other revegetation stages (Figure 5). This indicated that the climax forest possessed unique soil fungal communities among revegetation stages. The different soil locations of the pioneer weed, herbage, and shrub stages tended to group together (Figure 5), which implied that the soil fungal community compositions of these three revegetation stages were similar. The soil fungal community composition at the farmland stage was more similar to the shrub, pioneer weed, and herbage stages than the early and climax forests stages (Figure 5). Moreover, ANOSIM revealed that there was a significant statistical differences (Bray-Curtis – *R* = 0.9257, *p* = 0.001) of soil fungal communities at OTU-level among six revegetation stages (Supplementary Table S4). PERMANOVA showed that natural revegetation exhibited a strong effect (*R*^2^ = 0.5612; *p* = 0.001) on the soil fungal community variances at OTU-level (Supplementary Tables S5–S7).

### Vital drivers for the variations in soil fungal communities

**Figure 6:**
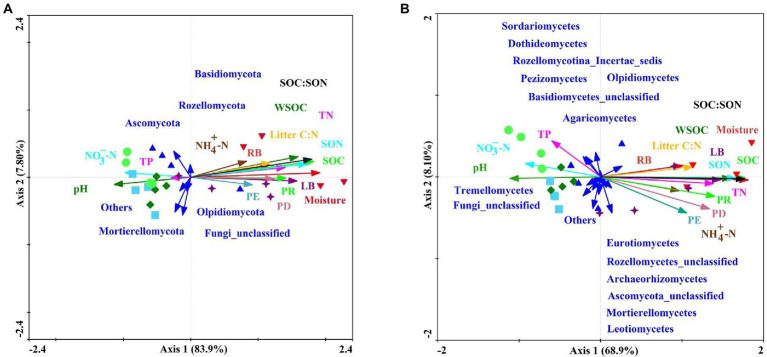
Redundancy analysis diagram illustrating the relationships between the compositions of soil fungal communities at the phylum-level **(A)** and class-level **(B)** from different sampling sites under variable environments. Explanatory variables are shown *via* different arrows: soil fungal community composition by solid dark blue arrows; and the environmental variables by colored arrows: Plant species richness (PR), plant species diversity (PD), plant species evenness (PE), litter biomass (LB), root biomass (RB), litter C:N, soil moisture, pH, soil organic carbon (SOC), soil water-soluble organic carbon (WSOC), total nitrogen (TN), soil organic nitrogen (SON), ammonium nitrogen (NH_4_^+^-N), nitrate nitrogen (NO_3_^−^-N), total phosphorus (TP), available phosphorus (AP), and SOC:SON. Red circles represent farmland (S1) soil; dark blue upward triangles represent pioneer weed (S2) soil; dark green diamonds represent herbage (S3) soil; yellow squares represent shrub (S4) soil; dark purple stars represent early forest (S5) soil; and red downward triangles represent climax forest (S6).

**Figure 7:**
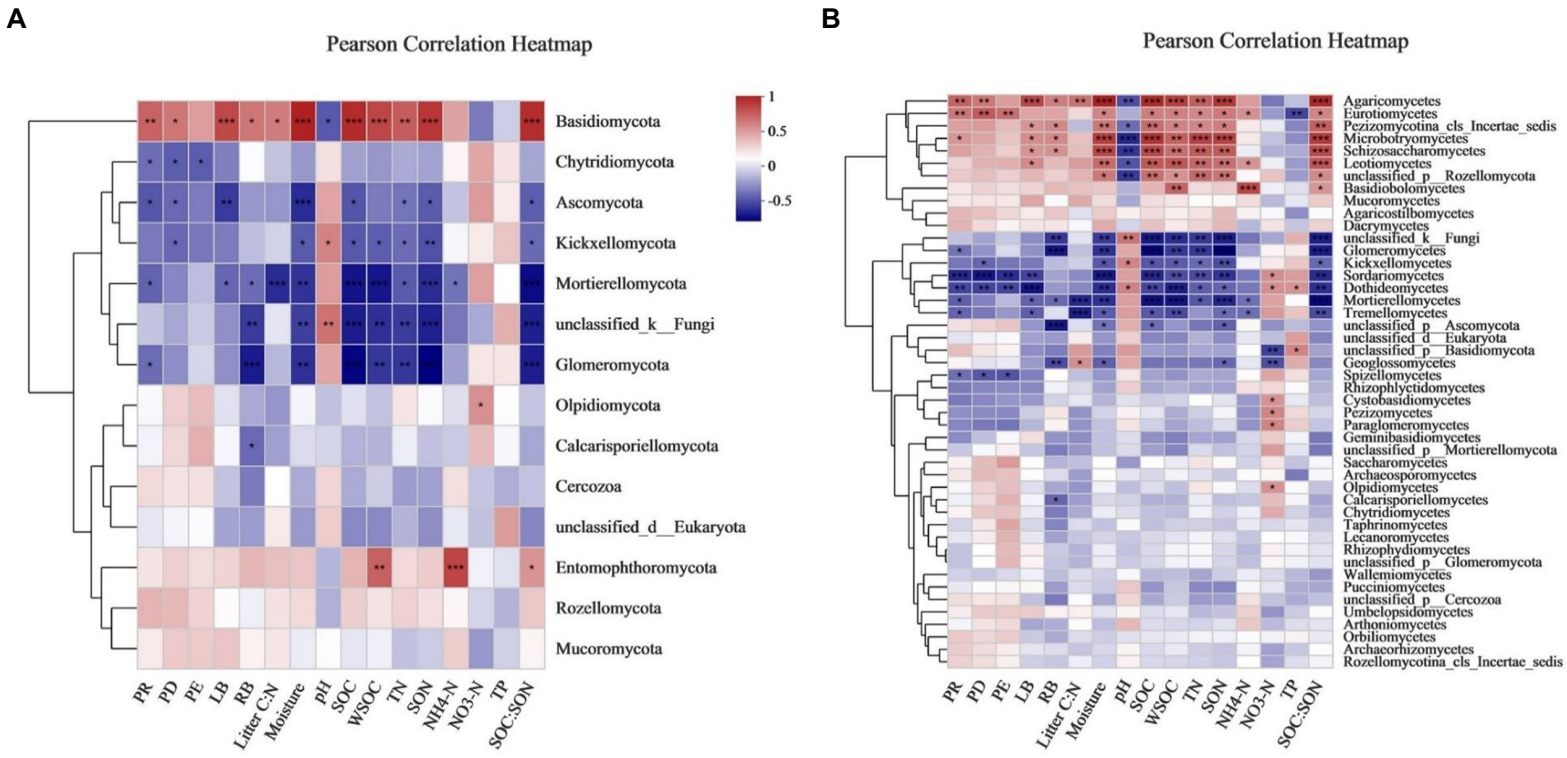
Heat map of the Pearson correlation between the bacterial **(A)** phyla and **(B)** class with environmental variables. Figure 6 for abbreviations.

Sixteen environmental variables (i.e., PR, PD, PE, LB, RB, litter C:N ratio, soil moisture, pH, SOC, WSOC, TN, SON, NH_4_^+^-N, NO_3_^−^-N, TP, and SOC:SON) explained 93.0 and 84.1% of the total changes in the soil fungal community compositions at the phylum and class levels, respectively (Figure 6). PERMANOVA revealed that variations in the soil fungal community composition at the phylum-and class-levels were both significantly affected by soil moisture, SOC, SON, SOC:SON, WSOC, LB, TN, PR, litter C:N ratio, PD, RB, pH, and NO_3_^−^-N (Supplementary Tables S6, S7). Pearson’s correlation analysis exhibited that variations in the soil fungal community abundance (i.e., ITS gene copy number) were strongly related to the LB, RB, soil moisture, SOC, WSOC, TN, SON, NH_4_^+^-N, and SOC:SON (Table 5). Species richness indices (i.e., ACE and Chao1) were intimately associated with plant richness, diversity, and evenness (Table 5). The Shannon diversity index was negatively correlated with LB, RB, litter C:N ratio, soil moisture, SOC, WSOC, TN, SON, and SOC:SON (Table 5). The relative abundance of *Ascomycota* was significantly negatively correlated with the PR, PD, LB, soil moisture, SOC, TN, SON, SOC:SON (Figure 7A). The relative abundances of *Basidiomycota*, and *Agaricomycetes* were strongly correlated with the PR, PD, LB, RB, litter C:N ratio, soil moisture, SOC, WSOC, TN, SON, and SOC:SON (Figures 7A,B). The relative abundances of *Eurotiomycetes, Leotiomycetes, Rozellomycota_unclassified, Microbotryomycctes, Schizosaccharomycetes* were strongly related to soil moisture, SOC, WSOC, TN, SON, SOC:SON (Figure 7B). The relative abundance of Symbiotroph was intimately related to LB, RB, SOC, WSOC, TN, SON, and SOC:SON (Table 5). The relative abundance of Saprotroph had a significantly negative correlation with the LB, RB, soil moisture, WSOC, and SOC:SON (Table 5). The relative abundance of Pathotroph was negative associated with the RB, soil moisture, SOC, and SON (Table 5). The relative abundances of Undefined fungi, AMF, and Fungal Parasites had a significantly negative correlation with the soil moisture, SOC, WSOC, TN, SON, and SOC:SON (Table 5).

**Table 5:** Pearson correlations coefficients between fungal abundance, diversity, trophic modes, guilds and the environmental variables (*n* = 4) among revegetation stages in the Loess Plateau of China.

	PR	PD	PE	LB	RB	Litter C:N	Moisture	pH	SOC	WSOC	TN	SON	NH_4_^+^-N	NO_3_^−^-N	TP	SOC:SON
Gene of ITS1-2 copies/g	0.329	0.346	0.263	0.497^*^	0.491^*^	0.382	0.637^**^	−0.461^*^	0.643^**^	0.790^**^	0.453^**^	0.547^**^	0.602^**^	−0.157	−0.183	0.730^**^
OTU richness	0.184	0.201	0.171	−0.421^*^	−0.277	−0.330	−0.233	0.119	−0.190	−0.261	−0.058	−0.085	−0.166	0.221	−0.177	−0.237
Richness (Ace)	0.511^*^	0.550^**^	0.505^*^	−0.146	−0.250	−0.135	0.016	0.009	0.028	−0.064	0.152	0.081	0.178	−0.103	−0.270	0.014
Richness (Chao1)	0.477^*^	0.523^**^	0.484^*^	−0.173	−0.257	−0.149	0.001	−0.004	0.024	−0.057	0.156	0.074	0.182	−0.077	−0.271	0.018
Shannon	−0.339	−0.161	−0.024	−0.514^*^	−0.437^*^	−0.577^**^	−0.563^**^	0.380	−0.606^**^	−0.627^**^	−0.379	−0.521^**^	−0.253	0.300	−0.114	−0.636^**^
Simpson	0.360	0.220	0.129	0.527^**^	0.289	0.438^*^	0.451^*^	−0.350	0.509^*^	0.447^*^	0.303	0.453^*^	0.201	−0.309	0.092	0.532^**^
Symbiotroph	0.343	0.376	0.317	0.785^**^	0.529^**^	0.239	0.736	−0.504^*^	0.694^**^	0.612^**^	0.481^*^	0.632^**^	0.325	−0.159	−0.216	0.732^**^
Saprotroph	−0.321	−0.223	−0.118	−0.484^*^	−0.417^*^	−0.340	−0.477^*^	0.271	−0.394	−0.463^**^	−0.233	−0.314	−0.229	0.135	−0.061	−0.437^*^
Pathotroph	−0.067	−0.166	−0.122	−0.266	−0.427^*^	0.029	−0.503^*^	0.355	−0.434^*^	−0.324	−0.401	−0.462^*^	−0.031	−0.148	0.368	−0.352
Pathotroph-saprotroph	0.022	−0.105	−0.161	−0.251	−0.175	−0.003	−0.283	0.214	−0.111	−0.230	−0.206	−0.055	−0.172	−0.202	−0.066	−0.161
Pathotroph-symbiotroph	0.414^*^	0.270	0.158	0.260	0.320	0.487^*^	0.641^**^	−0.439^*^	0.592^**^	0.584^**^	0.519^**^	0.546^**^	0.183	−0.299	−0.161	0.582^**^
Saprotroph-symbiotroph	0.494^*^	0.457^*^	0.327	0.211	0.211	0.562^**^	0.495^**^	−0.300	0.548^**^	0.494^*^	0.504^*^	0.537^**^	0.374	−0.299	0.036	0.492^*^
Pathotroph-saprotroph-symbiotroph	0.006	0.150	0.173	−0.030	−0.310	−0.009	−0.103	0.046	−0.262	−0.120	−0.253	−0.308	0.090	0.078	−0.323	−0.184
Undefined fungi	−0.576^**^	−0.510^*^	−0.404	−0.591^**^	−0.301	−0.589^**^	−0.684^**^	0.457^*^	−0.722^**^	−0.662^**^	−0.544^**^	−0.653^**^	−0.460^*^	0.450^*^	0.056	−0.758^**^
ECM	0.380	0.404	0.334	0.787^**^	0.539^**^	0.264	0.755^**^	−0.519^**^	0.714^**^	0.638^**^	0.496^*^	0.649^**^	0.337	−0.186	−0.225	0.752^**^
AMF	−0.433^*^	−0.328	−0.104	−0.321	−0.683^**^	−0.202	−0.623^**^	0.388	−0.752^**^	−0.600^**^	−0.601^**^	−0.771^**^	−0.277	0.107	0.114	−0.676^**^
Endophyte	−0.511^*^	−0.478^*^	−0.400	−0.293	−0.052	−0.445^*^	−0.317	0.173	−0.245	−0.381	−0.113	−0.186	−0.343	0.637^**^	0.259	−0.283
Dung saprotroph	−0.745^**^	−0.840^**^	−0.868^**^	−0.474^*^	0.203	−0.398	−0.408^*^	0.348	−0.386	−0.388	−0.365	−0.354	−0.401	0.611^**^	0.444^*^	−0.428^*^
Dung saprotroph-plant saprotroph	−0.637^**^	−0.681^**^	−0.683^**^	−0.474^*^	0.065	−0.449^*^	−0.430^*^	0.412^*^	−0.383	−0.413^*^	−0.348	−0.331	−0.453^*^	0.552^**^	0.461^*^	−0.437^*^
Dung saprotroph-soil saprotroph	−0.505^*^	−0.353	−0.101	−0.211	−0.238	−0.303	−0.331	−0.011	−0.404	−0.273	−0.236	−0.384	−0.384	0.355	0.167	−0.381
Undefined saprotroph	−0.110	0.055	0.176	−0.219	−0.339	−0.029	−0.186	0.042	−0.093	−0.224	0.065	−0.002	0.018	−0.016	−0.229	−0.172
Undefined saprotroph-wood saprotroph	−0.086	−0.141	−0.048	−0.123	−0.416^*^	−0.224	−0.309	0.214	−0.371	−0.247	−0.350	−0.358	−0.176	−0.006	0.075	−0.370
Wood saprotroph	0.137	0.071	0.003	−0.245	−0.029	0.095	0.087	−0.161	0.030	0.185	0.054	0.077	−0.126	0.150	−0.117	−0.001
Animal pathogen	0.073	−0.034	−0.038	−0.212	−0.267	0.134	−0.367	0.236	−0.265	−0.188	−0.286	−0.293	0.058	−0.214	0.332	−0.190
Plant pathogen	−0.379	−0.382	−0.248	−0.126	−0.429^*^	−0.277	−0.392	0.340	−0.493^*^	−0.355	−0.323	−0.484^*^	−0.251	0.147	0.154	−0.441^*^
Fungal parasite	−0.186	−0.052	0.045	−0.351	−0.615^**^	−0.097	−0.491^*^	−0.501^*^	−0.540^**^	−0.460^*^	−0.524^**^	−0.532^**^	−0.041	−0.098	−0.116	−0.511^*^

See Tables 1, 4 for abbreviations.

* *p* < 0.05;

** *p* < 0.01.

## Discussion

### Soil fungal abundance and diversity change along with natural revegetation

Our results revealed that the soil fungal abundance was greatly restored during the later stages of natural revegetation, notably in the climax forest in the Loess Plateau (Table 1). The soil fungal communities were co-regulated by plant and soil properties (Thompson et al., 2017; Mariotte et al., 2018). The quantity of plant litter and root inputs into the soil can directly impact soil nutrient substrates (e.g., SOC, WSOC, TN, SON, and NH_4_^+^-N), which have been widely considered as the primary driving factors for soil fungal abundance (Peay et al., 2013; Yang et al., 2019), due to numerous soil fungi being saprophytic (Zimudzi et al., 2018). In the present study, LB, RB, SOC, WSOC, TN, SON, and NH_4_^+^-N were gradually enhanced along with natural revegetation (Supplementary Table S2). Pearson’s correlation analysis confirmed that the soil fungal abundance was intimately related to the LB, RB, SOC, WSOC, TN, SON, and NH_4_^+^-N (Table 5). Hence, soil fungal abundance in the climax forest was the greatly enhanced, which was primarily attributed to high levels of plant biomass and soil nutrient substrates (Table 1; Supplementary Table S2). Furthermore, soil fungal communities can be affected by litter quality (Bachelot et al., 2016). It has been documented that soil fungi preferentially degrade recalcitrant plant materials (e.g., lignin and hemicellulose) into smaller molecules by producing extracellular enzymes (Voříšková et al., 2014). Generally, a high litter C:N ratio implies low litter quality (Yang et al., 2019), whereas a high SOC:SON ratio indicates low SOM decomposition (Artemyeva et al., 2021). In present study, the climax forest exhibited the highest litter C:N and SOC:SON ratios (Supplementary Table S2), and the greatest recalcitrance index for soil C among revegetation stages (Zhang et al., 2022). Accordingly, we deduced that a lower litter quality and more recalcitrant organic C in the climax forest may be instrumental for the flourishing of soil fungi, which prefer to degrade recalcitrant substrates (Supplementary Table S2; Voříšková et al., 2014; Yang et al., 2019).

Natural revegetation substantially altered soil fungal richness and diversity in the Loess Plateau of China (Table 1). Surprisingly, the soil fungal richness (i.e., OTU richness, ACE, and Chao1) did not continuously increase along with natural revegetation, which appeared first to rise and then decline at the climax forest stage (Table 1). Various plants provide distinct photosynthetic C for soil fungi, which allow plant communities to modify soil fungal communities; thus, affecting soil fungal richness and diversity (Wardle, 2006; Zuo et al., 2016). Notably, plant properties (e.g., species richness, diversity, and evenness) have been confirmed to greatly impact soil fungal richness and diversity (Peay et al., 2013; LeBlanc et al., 2015; Goodness et al., 2016). In general, greater plant richness results in the increase in soil fungal richness (Peay et al., 2013; LeBlanc et al., 2015), as litter diversity increases the diversity of available resources for soil fungi (Zuo et al., 2016). This was supported by our results which exhibited that the ACE and Chao1 indexes for soil fungal communities were highly related to plant richness, diversity, and evenness (Table 5). It was reasoned that the changes in soil fungal richness along with natural revegetation were primarily driven by plant properties, particularly plant richness (Tables 1; Supplementary Table S2; Peay et al., 2013; LeBlanc et al., 2015). Additionally, greatly declined fungal Shannon diversity index at the climax forest stage was likely resulted from distinct growth strategies of microorganisms (Table 1), where soil microbial population in the late stage of vegetation succession lives in predictable and stable environment, resulting in strong competitiveness (*K*-selected) and low fungal diversity (Zhang et al., 2016).

### Changes and driving forces of soil fungal community composition along with natural revegetation

Natural revegetation markedly influenced the composition of soil fungal communities (Supplementary Tables S5–S7; Figure 5). The PCoA and Bray–Curtis similarity indices exhibited that the composition of soil fungal communities gradually shifted along with natural revegetation, where the climax forest and farmland stages were both significantly distinct from the pioneer weed, herbage, and shrub stages, respectively (Figure 5). PERMANOVA clearly demonstrated that the total variations in the soil fungal community composition at the phylum-and class-levels were strongly driven by soil moisture, SOC, SON, SOC:SON, WSOC, LB, TN, PR, litter C:N ratio, PD, RB, pH, and NO_3_^−^-N (Supplementary Tables S6, S7). This further confirmed that soil moisture (Brockett et al., 2012; Urbanová et al., 2015), SOC, SON, SOC:SON, and WSOC (Goldmann et al., 2015; Maestre et al., 2015; Balami et al., 2021), LB and RB (Yang et al., 2020), PR and PD (Goodness et al., 2016), pH (Maestre et al., 2015), and NO_3_^−^-N (Huang et al., 2019) were the critical drivers for variations in soil fungal community composition. In current study, the sum of relative abundances of *Ascomycota* and *Basidiomycota* reached 72.39 to 95.90% across all revegetation stages (Table 2). This result aligned with earlier studies, which revealed that *Ascomycota* and *Basidiomycota* are the most ubiquitously dominant phyla in soil fungal communities (Bello et al., 2021; Llimós et al., 2021; Nuñez et al., 2021). There was an obvious transition at the phylum level which showing that the relative abundance of *Basidiomycota* steadily increased, while the relative abundance of *Ascomycota* gradually decreased along with natural revegetation (Table 2). Our results supported Nuñez et al. (2021) who reported that the relative abundances of *Ascomycota* and *Basidiomycota* may be an indicator of a succession trajectory, which may evaluate the progress and success of vegetation reconstruction, while the more representative *Ascomycete* might be an indicator of ecosystem degradation (Gourmelon et al., 2016).

Generally, *Ascomycota* and *Basidiomycota* have competitive intracolony relationships (Huang et al., 2022). *Ascomycota* are oligotrophic fungal groups (Clemmensen et al., 2015) that flourish under the low nutritional conditions (Yang et al., 2019). Whereas, *Basidiomycota* prefer copiotrophic environments (Sterkenburg et al., 2015). *Ascomycota* were gradually replaced by *Basidiomycota* along with natural revegetation had been greatly driven by steadily raised plant biomass (i.e., LB, RB), and soil nutrient substrates (e.g., SOC, WSOC, TN, SON, and NH_4_^+^-N; Table 2; Supplementary Table S2), which restricted the growth of oligotrophic *Ascomycota*, but were favored by copiotrophic *Basidiomycota* (Cho et al., 2017). Additionally, *Ascomycota* and *Basidiomycota* degrade large molecules, convert complex organic matter to available elements (Essel et al., 2018), and play crucial roles in litter and SOM decomposition (Baldrian et al., 2012; Xiao et al., 2022). *Ascomycota* have been reported to favor high-quality substrates (e.g., low C:N; Thomson et al., 2015), and possess a stronger capacity for the degradation of plant cellulose, hemicellulose, and simple substrates (e.g., sugar; Leung et al., 2016; Bello et al., 2021). While *Basidiomycota* are inclined to degrade low-quality substrates (e.g., high C:N; Thomson et al., 2015), and recalcitrant lignin and lignocellulose substrates (Llimós et al., 2021), which rely more on the decomposition of wood components to obtain C sources (Mali et al., 2017). LEfSe analyses and LDA values revealed that the most abundant members of *Ascomycota*, including *Sordariomycetes*, *Sordariales*, *Chaetomiaceae*, *Chaetomium*, *Dothideomycetes*, *Pleosporales*, *Nectriaceae*, *Didymellaceae*, *Sporormiaceae*, *Fusarium*, *Lasiosphaeriaceae*, *Epicoccum*, and *Preussia* were present at the farmland stage (Figures 3A,B). This was potentially attributed to the high-quality substrates derived from litter and soil (i.e., low ratios of litter C:N and SOC:SON; Thomson et al., 2015), as well as lower levels of LB, SOC, TN, and SON at the farmland stage relative to other revegetation stages (Supplementary Table S2). In terms of the temporal progression of revegetation, the most enriched members of *Basidiomycota*, including *Agaricomycetes*, *Agaricales*, *Cortinariaceae*, *Cortinarius*, *Sebacinales*, *Sebacinaceae*, *Tricholomataceae*, *Tricholoma*, *Russulales,* and *Russulaceae* were found at the climax forest stage (Figures 3A,B). This primarily resulted from low-quality substrates (e.g., high ratios of litter C:N, SOC:SON, and recalcitrant SOC; Supplementary Table S2; Thomson et al., 2015; Zhang et al., 2022), and higher levels of LB, RB, SOC, WSOC, TN, and SON at the climax forest stage compared with other revegetation stages (Supplementary Table S2). These inferences were further confirmed by the results of Pearson’s correlation analysis (Figure 7).

### Shifts in soil fungal trophic modes and functional groups along with natural revegetation

Natural revegetation significantly shifted soil fungal trophic modes and functional groups in the Loess Plateau of China (Table 4; Figure 4). Specifically, relative abundance of symbiotic fungi progressively increased along with natural revegetation (Figure 4) that primarily resulted from the contribution of ECM fungi (Table 4). ECM fungi ubiquitously exist in forest soils (Urbanová et al., 2015), particularly in boreal and temperate forests (Pellitier and Zak, 2021), and can be symbiotic with ~60% of trees worldwide (Steidinger et al., 2019; Anthony et al., 2022). ECM fungi assist with the early establishment of trees through the promotion of seedling growth for host plants (Bennett et al., 2017; Anthony et al., 2022); provide host plants with more N to satisfy their demands for N (Pellitier and Zak, 2021); and protect tree seedlings from pathogens (Anthony et al., 2022). ECM fungi are tremendously affected by disturbances (Bermúdez-Contreras et al., 2022), as evidenced by previous studies, reporting that anthropogenic disturbances can result in decreased ECM fungi propagules and hyphal networks, which can serve as inoculum for new seedlings (Rodriguez-Ramos et al., 2021; Bermúdez-Contreras et al., 2022). It was inferred that ECM fungi were most enriched at the climax forest stage (Table 4), which may have been primarily due to ECM fungi having a partiality for symbioses with dominant woody plants in forests, in contrast to shrubs, herbs, and farmland (Urbanová et al., 2015; Pellitier and Zak, 2021). Furthermore, human disturbances gradually declined along with natural revegetation, which promoted the prosperity of ECM fungi propagules and hyphal network (Table 4; Bermúdez-Contreras et al., 2022). Interestingly, AMF and endophyte fungi were not observed, or were very scarce, at the climax forest stage, which were enriched in the early stages of revegetation (Table 4). AMF and endophyte fungi have been extensively reported to play central roles in enhancing host plant resistance and/or tolerance to various stresses (e.g., drought, pathogens; Fall et al., 2021), while promoting the growth of host plants through N fixation, the mobilization of nutrients, and phosphate solubilization (Coban et al., 2022; Yan et al., 2022). Thus, we reasoned that the most enriched AMF and endophyte fungi in the early stages of revegetation would facilitate the growth of host plants. They would also enhance the resistance and/or tolerance of farmland, pioneer weed, herbage, and shrub to abundant pathotrophic fungi and harsh environments (e.g., drought) during the early stages of revegetation (Supplementary Tables S2, S4; Figure 4).

In current study, relative abundance of saprotrophic fungi progressively decreased along with natural revegetation (Figure 4). Previous studies clarified that saprotrophic fungi dominated in the litter layer, which had fundamental niches with ECM fungi, and were less abundant in woodlands (Bödeker et al., 2016). Li et al. (2022) documented that ECM fungi could inhibit the activities of saprotrophic fungi by regulating litter decomposition, leading to the reduced abundance of saprotrophic fungi. Generally, after the loss of ECM fungi, saprotrophic fungi may become dominant (Li et al., 2022). Human disturbances have been reported to shift the dominance from ECM to saprophytic fungi and induce an increase in saprophytic fungi (Rodriguez-Ramos et al., 2021; Li et al., 2022). It was inferred that saprotrophic fungi were most enriched at the pioneer weed stage (Figure 4), which may have been attributed to strong human disturbances during the initial stage of natural revegetation. This would be detrimental to ECM fungi, and promote the flourishing of saprotrophic fungi by relieving the suppression of ECM on saprotrophic fungi (Rodriguez-Ramos et al., 2021; Li et al., 2022). Saprotrophic fungi have been broadly recognized as vital fungal groups that regulate nutrient cycles in terrestrial ecosystems by accelerating the decomposition of dead plant materials and SOM (Bello et al., 2021; Hagenbo et al., 2022), which enables them and other microorganisms to obtain and utilize nutrients (Maranón-Jiménez et al., 2021). We reasoned that lower levels of SOC and SON at the pioneer weed stage resulted primarily from lower plant residue inputs (i.e., litter and roots; Supplementary Table S2), and the higher enrichment of saprotrophic fungi that expedited SOM decomposition, in contrast to the climax forest (Figure 4; Hagenbo et al., 2022). Prior investigations revealed that symbiotic fungi boosted recalcitrant C sequestration due to symbiotic fungi, particularly ECM fungi and AMF that contained recalcitrant compounds (e.g., chitin and glomalin, respectively; Godbold et al., 2006; Hagenbo et al., 2022). Mycorrhizal hyphal biomass and turnover comprise the most important C source inputs into the soil, which facilitate the accumulation of soil C (Godbold et al., 2006; Hagenbo et al., 2022). It was extrapolated that the most enriched symbiotic fungi (especially ECM fungi) were in the climax forest, as vital C inputs likely significantly contributed to the greatest SOC and SON sequestration (Supplementary Tables S2, S4; Figure 4).

Pathotrophic fungi may acquire nutrients by attacking host cells (Wutkowska et al., 2019). In present study, the relative abundance of pathotrophic fungi was highest at the pioneer weed stage, which greatly declined in the late stages of revegetation (Figure 4). Desprez-Loustau et al. (2006) showed that pathotrophic fungi can thrive under the condition that the water potential is far lower than the minimum water potential required for plant growth. We found that pathotrophic fungi exhibited a significantly negative correlation with soil moisture (Table 5), which was corroborated by Hagenbo et al. (2022). Generally, drought-induced diseases are typically triggered by endophytic potential pathogenic fungi (Hagenbo et al., 2022). We inferred that increased pathotrophic fungi in the early stages of revegetation might be partially driven by lower soil moisture relative to the late stages of revegetation (Supplementary Table S2; Figure 4). The greatly decreased pathotrophic fungi in the late stages of revegetation implied that natural revegetation processes reduced the risk of fungal diseases (Figure 4; Hu et al., 2022). This might be associated with the considerably increased ECM fungi that protect trees from pathogens at the early and climax forest stages (Table 4; Anthony et al., 2022).

## Conclusion

In summary, our results demonstrated that ~160 years of natural revegetation greatly enhanced soil fungal abundance during the later stages of revegetation. However, soil fungal richness did not continuously increase along with natural revegetation and decreased at the climax forest stage. The lowest soil fungal diversity was found in the climax forest. Soil fungal community compositions gradually changed from oligotrophic *Ascomycota* to copiotrophic *Basidiomycota* along with natural revegetation, which also significantly shaped soil fungal trophic modes and functional groups. Specifically, the relative abundances of symbiotic fungi progressively raised, while saprotrophic and Pathotrophic fungi gradually declined along with natural revegetation. The symbiotic fungi (particularly ECM fungi that contained more recalcitrant compounds) that increased greatly in the climax forest, which as vital C sources were conducive to SOC and SON sequestration in the climax forest. The higher enrichment of saprotrophic fungi during the early stages of revegetation expedited the decomposition of SOM, and decreased SOC and SON sequestration, in contrast to the climax forest. Alterations in soil fungal abundance, diversity, community composition, trophic modes and functional groups were primarily driven by variations in plant properties (e.g., plant species richness, diversity, evenness, litter quantity and quality), and the quantity (i.e., concentration) and quality (e.g., SOC:SON) levels of soil nutrient substrates, soil moisture and pH. These findings provide insight into the variations and driving mechanism behind changes in soil fungal communities following natural revegetation, which have implications for evaluating effectiveness of ecological rehabilitation strategies from the perspective of soil microbial recovery.

## Data availability statement

The data presented in the study are deposited in the Sequence Read Archive (SRA) database of the National Center for Biotechnology Information (NCBI) repository, accession number SRP388698.

## Author contributions

WY performed the experiment, analyzed the data, and drafted the manuscript. LD, YW, XY, and HZ participated in the main experiments. JW, YL, and SA reviewed the manuscript and contributed to revisions. WY and XC designed the study and revised the manuscript. All authors contributed to the article and approved the submitted version.

## Funding

This study was supported by the National Natural Science Foundation of China (grant no. 32071632; 31600427), the Natural Science Foundation of Shaanxi Province, China (grant no. 2022JM-114; 2019JQ-666), and the Fundamental Research Funds for the Central Universities (grant no. GK202003051).

## Conflict of interest

The authors declare that the research was conducted in the absence of any commercial or financial relationships that could be construed as a potential conflict of interest.

## Publisher’s note

All claims expressed in this article are solely those of the authors and do not necessarily represent those of their affiliated organizations, or those of the publisher, the editors and the reviewers. Any product that may be evaluated in this article, or claim that may be made by its manufacturer, is not guaranteed or endorsed by the publisher.

## Footnotes

^1^ http://canoco.software.informer.com/4.5/

^2^ https://cran-archive.r-project.org/bin/windows/base/old/3.2.2/

## References

[ref400] Abarenkov K. Nilsson R. H. Larsson K. H. Alexander I. J. Eberhardt U. Erland S.. (2010). The UNITE database for molecular identification of fungi–recent updates and future perspectives. New Phytol. 186, 281–285. doi: 10.1111/j.1469-8137.2009.03160.x, PMID: 20409185

[ref1] Anderson M. J. Walsh D. C. (2013). PERMANOVA, ANOSIM, and the mantel test in the face of heterogeneous dispersions: what null hypothesis are you testing? Ecol. Monogr. 83, 557–574. doi: 10.1890/12-2010.1

[ref2] Anthony M. A. Crowther T. W. van der Linde S. Suz L. M. Bidartondo M. I. Cox F.. (2022). Forest tree growth is linked to mycorrhizal fungal composition and function across Europe. ISME J. 16, 1327–1336. doi: 10.1038/s41396-021-01159-7, PMID: 35001085 PMC9038731

[ref3] Artemyeva Z. Danchenko N. Kolyagin Y. Kirillova N. Kogut B. (2021). Chemical structure of soil organic matter and its role in aggregate formation in Haplic Chernozem under the contrasting land use variants. Gatena 204, 105403. doi: 10.1016/j.catena.2021.105403

[ref4] Bachelot B. Uriarte M. Zimmerman J. Thompson J. Leff J. W. Asiaii A.. (2016). Long-lasting effects of land use history on soil fungal communities in second-growth tropical rain forests. Ecol. Appl. 26, 1881–1895. doi: 10.1890/15-1397.1, PMID: 27755697

[ref5] Balami S. Vašutová M. Košnar J. Karki R. Khadka C. Tripathi G.. (2021). Soil fungal communities in abandoned agricultural land has not yet moved towards the seminatural forest. For. Ecol. Manag. 491, 119181. doi: 10.1016/j.foreco.2021.119181

[ref6] Baldrian P. Kolařík M. Štursová M. Kopecký J. Valášková V. Větrovský T.. (2012). Active and total microbial communities in forest soil are largely different and highly stratified during decomposition. ISME J. 6, 248–258. doi: 10.1038/ismej.2011.95, PMID: 21776033 PMC3260513

[ref8] Bello A. Wang B. Zhao Y. Yang W. Ogundeji A. (2021). Composted biochar affects structural dynamics, function and co-occurrence network patterns of fungi community. Sci. Total Environ. 775, 145672. doi: 10.1016/j.scitotenv.2021.145672, PMID: 33618307

[ref9] Bennett J. A. Maherali H. Reinhart K. O. Lekberg Y. Hart M. M. Klironomos J. (2017). Plant-soil feedbacks and mycorrhizal type influence temperate forest population dynamics. Science 355, 181–184. doi: 10.1126/science.aai8212, PMID: 28082590

[ref10] Bermúdez-Contreras A. I. Monroy-Guzmán C. Pérez-Lucas L. Escutia-Sánchez J. A. Del Olmo-Ruiz M. Truong C. (2022). Mycorrhizal fungi associated with juniper and oak seedlings along a disturbance gradient in Central Mexico. Front. For. Glob. Change 5, 736664. doi: 10.3389/ffgc.2022.736664

[ref11] Bödeker I. T. M. Lindahl B. D. Olson A. Clemmensen K. E. (2016). Mycorrhizal and saprotrophic fungal guilds compete for the same organic substrates but affect decomposition differently. Funct. Ecol. 30, 1967–1978. doi: 10.1111/1365-2435.12677

[ref12] Brockett B. F. T. Prescott C. E. Grayston S. J. (2012). Soil moisture is the major factor influencing microbial community structure and enzyme activities across seven biogeoclimatic zones in western Canada. Soil Biol. Biochem. 44, 9–20. doi: 10.1016/j.soilbio.2011.09.003

[ref14] Cai X. W. Zhang D. Wang Y. Q. Diao L. F. Cheng X. L. Luo Y. Q.. (2022). Shift in soil microbial communities along ~160 years of natural vegetation restoration on the Loess Plateau of China. Appl. Soil Ecol. 173, 104394. doi: 10.1016/j.apsoil.2022.104394

[ref15] Campbell J. E. Lobell D. B. Genova R. C. Field C. B. (2008). The global potential of bioenergy on abandoned agriculture lands. Environ. Sci. Technol. 42, 5791–5794. doi: 10.1021/es800052w, PMID: 18754510

[ref16] Chen C. (1954). The vegetation and its roles in soil and water conservation in the secondary forest area in the boundary of Shaanxi and Gansu provinces. Acta Phytoecol. Geobot. Sin. 2, 152–153.

[ref17] Cho H. Kim M. Tripathi B. Adams J. (2017). Changes in soil fungal community structure with increasing disturbance frequency. Microb. Ecol. 74, 62–77. doi: 10.1007/s00248-016-0919-1, PMID: 28062901

[ref18] Clemmensen K. E. Finlay R. D. Dahlberg A. Stenlid J. Wardle D. A. Lindahl B. D. (2015). Carbon sequestration is related to mycorrhizal fungal community shifts during long-term succession in boreal forests. New Phytol. 205, 1525–1536. doi: 10.1111/nph.13208, PMID: 25494880

[ref19] Coban O. De Deyn G. B. van der Ploeg M. (2022). Soil microbiota as game-changers in restoration of degraded lands. Science 375, 990. doi: 10.1126/science.abe0725 35239372

[ref20] Crouzeilles R. Curran M. Ferreira M. S. Lindenmayer D. B. Grelle C. E. V. Benayas J. M. R. (2016). A global meta-analysis on the ecological drivers of forest restoration success. Nat. Commun. 7, 11666. doi: 10.1038/ncomms11666, PMID: 27193756 PMC4874030

[ref21] Deng L. Liu G. B. Shuangguan Z. P. (2014). Land-use conversion and changing soil carbon stocks in China’s ‘grain-for-green’ program: a synthesis. Glob. Chang. Biol. 20, 3544–3556. doi: 10.1111/gcb.12508, PMID: 24357470

[ref22] Desprez-Loustau M.-L. Marçais B. Nageleisen L.-M. Piou D. Vannini A. (2006). Interactive effects of drought and pathogens in forest trees. Ann. For. Sci. 63, 597–612. doi: 10.1051/forest:2006040

[ref23] Essel E. Li L. Deng C. Xie J. Zhang R. Luo Z.. (2018). Evaluation of bacterial and fungal diversity in a long-term spring wheat-field pea rotation field under different tillage practices. Can. J. Soil Sci. 98, 619–637. doi: 10.1139/cjss-2017-0155

[ref24] Fall F. Sanguin H. Fall D. Tournier E. Bakhoum N. Ndiaye C.. (2021). Changes in intraspecific diversity of the arbuscular mycorrhizal community involved in plant-plant interactions between *Sporobolus robustus* Kunth and *Prosopis juliflora* (Swartz) DC along an environmental gradient. Microb. Ecol. 83, 886–898. doi: 10.1007/s00248-021-01779-8 34245330

[ref25] Fierer N. Wood S. A. Bueno de Mesquita C. P. (2021). How microbes can, and cannot, be used to assess soil health. Soil Biol. Biochem. 153, 108111. doi: 10.1016/j.soilbio.2020.108111

[ref26] Fu B. Wang S. Liu Y. Liu J. Liang W. Miao C. (2017). Hydrogeomorphic ecosystem responses to natural and anthropogenic changes in the Loess Plateau of China. Annu. Rev. Earth Planet. Sci. 45, 223–243. doi: 10.1146/annurev-earth-063016-020552

[ref27] Geml J. Pastor N. Fernandez L. Pacheco S. Semenova T. A. Becerra A. G.. (2014). Large-scale fungal diversity assessment in the Andean Yungas forests reveals strong community turnover among forest types along an altitudinal gradient. Mol. Ecol. 23, 2452–2472. doi: 10.1111/mec.12765, PMID: 24762095

[ref28] Godbold D. L. Hoosbeek M. R. Lukac M. Cotrufo M. F. Janssens I. A. Ceulemans R.. (2006). Mycorrhizal hyphal turnover as a dominant process for carbon input into soil organic matter. Plant Soil 281, 15–24. doi: 10.1007/s11104-005-3701-6

[ref29] Goldmann K. Schöning I. Buscot F. Wubet T. (2015). Forest management type influences diversity and community composition of soil fungi across temperate forest ecosystems. Front. Microbiol. 6, 1300. doi: 10.3389/fmicb.2015.01300 26635766 PMC4656839

[ref30] Goodness J. Andersson E. Anderson P. M. Elmqvist T. (2016). Exploring the links between functional traits and cultural ecosystem services to enhance urban ecosystem management. Ecol. Indic. 70, 597–605. doi: 10.1016/j.ecolind.2016.02.031

[ref31] Gourmelon V. Maggia L. Powell J. R. Gigante S. Hortal S. Gueunier C.. (2016). Environmental and geographical factors structure soil microbial diversity in new Caledonian ultramafic substrates: a metagenomic approach. PLoS One 11:e0167405. doi: 10.1371/journal.pone.0167405, PMID: 27907121 PMC5131939

[ref32] Guo Y. Q. Hou L. J. Zhang Z. Y. Zhang J. L. Cheng J. M. Wei G. H.. (2019). Soil microbial diversity during 30 years of grassland restoration on the Loess Plateau, China: tight linkages with plant diversity. Land Degrad. Dev. 30, 1172–1182. doi: 10.1002/ldr.3300

[ref401] Gardes M. Bruns T. D. (1993). ITS primers with enhanced specificity for basidiomycetes_application to the identification of mycorrhizae and rusts. Mol. Ecol. 2, 113–118. doi: 10.1111/j.1365-294X.1993.tb00005.x, PMID: 8180733

[ref33] Hagenbo A. Alday J. G. de Aragon J. M. Castano C. De-Miguel S. Bonet J. A. (2022). Variations in biomass of fungal guilds are primarily driven by factors related to soil conditions in Mediterranean *Pinus pinaster* forests. Biol. Fertil. Soils 58, 487–501. doi: 10.1007/s00374-022-01621-4

[ref34] Hanif M. A. Guo Z. M. Moniruzzaman M. He D. Yu Q. S. Rao X. Q.. (2019). Plant taxonomic diversity better explains soil fungal and bacterial diversity than functional diversity in restored forest ecosystems. Plan. Theory 8, 479. doi: 10.3390/plants8110479, PMID: 31698841 PMC6918236

[ref35] Hu L. N. Li Q. Yan J. H. Liu C. Zhong J. X. (2022). Vegetation restoration facilitates belowground microbial network complexity and recalcitrant soil organic carbon storage in Southwest China karst region. Sci. Total Environ. 820, 153137. doi: 10.1016/j.scitotenv.2022.153137, PMID: 35041964

[ref36] Huang F. Y. Liu Z. H. Mou H. Y. Zhang P. Jia Z. K. (2019). Effects of different long-term farmland mulching practices on the loessial soil fungal community in a semiarid region of China. Appl. Soil Ecol. 137, 111–119. doi: 10.1016/j.apsoil.2019.01.014

[ref37] Huang C. J. Wu X. Q. Liu X. Y. Fang Y. T. Liu L. Wu C. S. (2022). Functional fungal communities dominate wood decomposition and are modified by wood traits in a subtropical forest. Sci. Total Environ. 806, 151377. doi: 10.1016/j.scitotenv.2021.151377, PMID: 34740660

[ref38] Khomich M. Davey M. L. Kauserud H. Rasconi S. Andersen T. (2017). Fungal communities in Scandinavian lakes along a longitudinal gradient. Fungal Ecol. 27, 36–46. doi: 10.1016/j.funeco.2017.01.008

[ref39] Khorchani M. Nadal-Romero E. Lasanta T. Tague C. (2022). Carbon sequestration and water yield tradeoffs following restoration of abandoned agricultural lands in Mediterranean mountains. Environ. Res. 207, 112203. doi: 10.1016/j.envres.2021.112203, PMID: 34648763

[ref40] Krishna M. Gupta S. Delgado-Baquerizo M. Morriën E. Garkoti S. C. Chaturvedi R.. (2020). Successional trajectory of bacterial communities in soil are shaped by plant-driven changes during secondary succession. Sci. Rep. 10, 9864. doi: 10.1038/s41598-020-66638-x, PMID: 32555419 PMC7299987

[ref41] Laforestlapointe I. Paquette A. Messier C. Kembel S. W. (2017). Leaf bacterial diversity mediates plant diversity and ecosystem function relationships. Nature 546, 145–147. doi: 10.1038/nature22399, PMID: 28538736

[ref42] LeBlanc N. L. Kinkel L. L. Kistler H. C. (2015). Soil fungal communities respond to grassland plant community richness and soil edaphics. Microb. Ecol. 70, 188–195. doi: 10.1007/s00248-014-0531-1, PMID: 25399511

[ref43] Leung H. T. C. Maas K. R. Wilhelm R. C. Mohn W. W. (2016). Long-term effects of timber harvesting on hemicellulolytic microbial populations in coniferous forest soils. ISME J. 10, 363–375. doi: 10.1038/ismej.2015.118, PMID: 26274049 PMC4737928

[ref44] Li J. Li S. F. Huang X. B. Tang R. Zhang R. Li C.. (2022). Plant diversity and soil properties regulate the microbial community of monsoon evergreen broad-leaved forest under different intensities of woodland use. Sci. Total Environ. 821, 153565. doi: 10.1016/j.scitotenv.2022.153565, PMID: 35101489

[ref45] Liu G. Y. Chen L. L. Shi X. R. Yuan Z. Y. Yuan L. Y. Lock T. R.. (2019). Changes in rhizosphere bacterial and fungal community composition with vegetation restoration in planted forests. Land Degrad. Dev. 30, 1147–1157. doi: 10.1002/ldr.3275

[ref46] Llimós M. Segarra G. Sancho-Adamson M. Trillas M. I. Romanyà J. (2021). Impact of olive saplings and organic amendments on soil microbial communities and effects of mineral fertilization. Front. Microbiol. 12, 653027. doi: 10.3389/fmicb.2021.653027, PMID: 34140935 PMC8203829

[ref47] Maestre F. T. Delgado-Baquerizo M. Jeffries T. C. Eldridge D. J. Ochoa V. Gozalo B.. (2015). Increasing aridity reduces soil microbial diversity and abundance in global drylands. Proc. Natl. Acad. Sci. U.S.A. 112, 15684–15689. doi: 10.1073/pnas.1516684112, PMID: 26647180 PMC4697385

[ref48] Mali T. Kuuskeri J. Shah F. Lundell T. K. (2017). Interactions affect hyphal growth and enzyme profiles in combinations of coniferous wood-decaying fungi of *Agaricomycetes*. PLoS One 12:e0185171. doi: 10.1371/journal.pone.0185171, PMID: 28953947 PMC5617175

[ref49] Maranón-Jiménez S. Radujković D. Verbruggen E. Grau O. Cuntz M. Peñuelas J.. (2021). Shifts in the abundances of saprotrophic and ectomycorrhizal fungi with altered leaf litter inputs. Front. Plant Sci. 12, 1452. doi: 10.3389/fpls.2021.682142 PMC8336600 34367207

[ref50] Mariotte P. Mehrabi Z. Bezemer T. M. De Deyn G. B. Kulmatiski A. Drigo B. (2018). Plant-soil feedback: bridging natural and agricultural sciences. Trends Ecol. Evol. 33, 129–142. doi: 10.1016/j.tree.2017.11.005, PMID: 29241940

[ref51] Niwaxu A. Shackleton C. M. (2019). The availability of non-timber forest products under forest succession on abandoned fields along the Wild Coast South Africa. Forests 10:1093. doi: 10.3390/f10121093

[ref580] Nguyen N. H. Song Z. W. Bates S. T. Branco S. Tedersoo L. Menke J.. (2016). FUNGuild: An open annotation tool for parsing fungal community datasets by ecological guild. Fungal Ecol. 20, 241–248. doi: 10.1016/j.funeco.2015.06.006, PMID: 31092941

[ref52] Nuñez N. F. Maggia L. Stenger P. Lelievre M. Letellier K. Gigante S.. (2021). Potential of high-throughput eDNA sequencing of soil fungi and bacteria for monitoring ecological restoration in ultramafic substrates: The case study of the new Caledonian biodiversity hotspot. Ecol. Eng. 173, 106416. doi: 10.1016/j.ecoleng.2021.106416

[ref53] Peay K. G. Baraloto C. Fine P. V. (2013). Strong coupling of plant and fungal community structure across western Amazonian rainforests. ISME J. 7, 1852–1861. doi: 10.1038/ismej.2013.66, PMID: 23598789 PMC3749505

[ref54] Pellitier P. T. Zak D. R. (2021). Ectomycorrhizal fungal decay traits along a soil nitrogen gradient. New Phytol. 232, 2152–2164. doi: 10.1111/nph.17734, PMID: 34533216

[ref55] Poole P. Ramachandran V. Terpolilli J. (2018). Rhizobia: from saprophytes to endosymbionts. Nat. Rev. Microbiol. 16, 291–303. doi: 10.1038/nrmicro.2017.171, PMID: 29379215

[ref56] Rodriguez-Ramos J. C. Cale J. A. Cahill J. F. Simard S. W. Karst J. Erbilgin N. (2021). Changes in soil fungal community composition depend on functional group and forest disturbance type. New Phytol. 229, 1105–1117. doi: 10.1111/nph.16749, PMID: 32557647

[ref57] Rosenzweig S. T. Carson M. A. Baer S. G. Blair J. M. (2016). Changes in soil properties, microbial biomass, and fluxes of C and N in soil following post-agricultural grassland restoration. Appl. Soil Ecol. 100, 186–194. doi: 10.1016/j.apsoil.2016.01.001

[ref581] Schloss P. D. Westcott S. L. Ryabin T. Hall J. R. Hartmann M. Hollister E. B.. (2009). Introducing mothur: open-source, platform-independent, community-supported software for describing and comparing microbial communities. Appl. Environ. Microb. 75, 7537–7541. doi: 10.1128/AEM.01541-09, PMID: 19801464 PMC2786419

[ref58] Steidinger B. S. Crowther T. W. Liang J. Nuland M. E. V. Werner G. D. A. Reich P. B.. (2019). Climatic controls of decomposition drive the global biogeography of forest-tree symbioses. Nature 569, 404–408. doi: 10.1038/s41586-019-1128-0, PMID: 31092941

[ref59] Sterkenburg E. Bahr A. Durling M. B. Clemmensen K. E. Lindal B. (2015). Changes in fungal communities along a boreal forest soil fertility gradient. New Phytol. 207, 1145–1158. doi: 10.1111/nph.13426, PMID: 25952659

[ref60] Sullivan B. W. Nifong R. L. Nasto M. K. Alvarez-Clare S. Dencker C. M. Soper F. M.. (2019). Biogeochemical recuperation of lowland tropical forest during succession. Ecology 100:e02641. doi: 10.1002/ecy.2641 30712256

[ref61] Tedersoo L. Bahram M. Polme S. Koljalg U. Yorou N. S. Wijesundera R.. (2014). Global diversity and geography of soil fungi. Science 346, 1256688. doi: 10.1126/science.1256688 25430773

[ref62] Thompson L. R. Sanders J. G. Mcdonald D. Amir A. Ladau J. Locey K. J. (2017). A communal catalogue reveals earth’s multiscale microbial diversity. Nature 551, 457–463. doi: 10.1038/nature24621, PMID: 29088705 PMC6192678

[ref63] Thomson B. C. Tisserant E. Plassart P. Uroz S. Griffiths R. I. Hannula S. E.. (2015). Soil conditions and land use intensification effects on soil microbial communities across a range of European field sites. Soil Biol. Biochem. 88, 403–413. doi: 10.1016/j.soilbio.2015.06.012

[ref64] Treseder K. K. Lennon J. T. (2015). Fungal traits that drive ecosystem dynamics on land. Microbiol. Mol. Biol. Rev. 79, 243–262. doi: 10.1128/MMBR.00001-15, PMID: 25971588 PMC4429240

[ref65] Urbanová M. Šnajdr J. Baldrian P. (2015). Composition of fungal and bacterial communities in forest litter and soil is largely determined by dominant trees. Soil Biol. Biochem. 84, 53–64. doi: 10.1016/j.soilbio.2015.02.011

[ref66] Voříšková J. Brabcová V. Cajthaml T. Baldrian P. (2014). Seasonal dynamics of fungal communities in a temperate oak forest soil. New Phytol. 201, 269–278. doi: 10.1111/nph.12481, PMID: 24010995

[ref67] Wall C. B. Egan C. P. Swift S. O. Hynson N. A. (2020). Three decades post-reforestation has not led to the reassembly of arbuscular mycorrhizal fungal communities associated with remnant primary forests. Mol. Ecol. 29, 4234–4247. doi: 10.1111/mec.15624, PMID: 32885507

[ref68] Wang J. Zhao W. W. Zhang X. Liu Y. Wang S. Liu Y. X. (2019). Effects of reforestation on plant species diversity on the Loess Plateau of China: a case study in Danangou catchment. Sci. Total Environ. 651, 979–989. doi: 10.1016/j.scitotenv.2018.09.266, PMID: 30257235

[ref69] Wardle D. A. (2006). The influence of biotic interactions on soil biodiversity. Ecol. Lett. 9, 870–886. doi: 10.1111/j.1461-0248.2006.00931.x 16796577

[ref70] Wutkowska M. Vader A. Mundra S. Cooper E. J. Eidesen P. B. (2019). Dead or alive; or does it really matter? Level of congruency between trophic modes in total and active fungal communities in high arctic soil. Front. Microbiol. 9, 3243. doi: 10.3389/fmicb.2018.03243, PMID: 30671045 PMC6333106

[ref71] Xiao D. He X. Y. Zhang W. Hu P. P. Sun M. M. Wang K. L. (2022). Comparison of bacterial and fungal diversity and network connectivity in karst and non-karst forests in Southwest China. Sci. Total Environ. 822, 153179. doi: 10.1016/j.scitotenv.2022.153179, PMID: 35051465

[ref72] Xu G. C. Cheng S. D. Li P. Li Z. B. Gao H. D. Yu K. X.. (2018). Soil total nitrogen sources on dammed farmland under the condition of ecological construction in a small watershed on the Loess Plateau, China. Ecol. Eng. 121, 19–25. doi: 10.1016/j.ecoleng.2017.09.005

[ref73] Xu M. P. Gao D. X. Fu S. Y. Lu X. Q. Wu S. J. Han X. H.. (2020). Long-term effects of vegetation and soil on the microbial communities following afforestation of farmland with *Robinia pseudoacacia* plantations. Geoderma 367, 114263. doi: 10.1016/j.geoderma.2020.114263

[ref740] Yan B. S. Sun L. P. Li J. J. Liang C. Q. Wei F. R. Xue S.. (2020). Change in composition and potential functional genes of soil bacterial and fungal communities with secondary succession in Quercus liaotungensis forests of the Loess Plateau, western China. Geoderma 364:114199. doi: 10.1016/j.geoderma.2020.114199, PMID: 35295292

[ref74] Yan K. Pei Z. Meng L. Zheng Y. Wang L. Feng R.. (2022). Determination of community structure and diversity of seed-vectored endophytic fungi in *Alpinia zerumbet*. Front. Microbiol. 13, 814864. doi: 10.3389/fmicb.2022.814864, PMID: 35295292 PMC8918987

[ref75] Yang T. Adams J. M. Shi Y. He J. S. Jing X. Chen L. T.. (2017). Soil fungal diversity in natural grasslands of the Tibetan plateau: associations with plant diversity and productivity. New Phytol. 215, 756–765. doi: 10.1111/nph.14606, PMID: 28542845

[ref76] Yang Y. Cheng H. Dou Y. X. An S. S. (2020). Plant and soil traits driving soil fungal community due to tree plantation on the Loess Plateau. Sci. Total Environ. 708, 134560. doi: 10.1016/j.scitotenv.2019.134560, PMID: 31780176

[ref77] Yang W. Yan Y. E. Jiang F. Leng X. Cheng X. L. An S. Q. (2016). Response of the soil microbial community composition and biomass to a short-term *Spartina alterniflora* invasion in a coastal wetland of eastern China. Plant Soil 408, 443–456. doi: 10.1007/s11104-016-2941-y

[ref78] Yang W. Zhang D. Cai X. W. Xia L. Luo Y. Q. Cheng X. L.. (2019). Significant alterations in soil fungal communities along a chronosequence of *Spartina alterniflora* invasion in a Chinese Yellow Sea coastal wetland. Sci. Total Environ. 693, 133548. doi: 10.1016/j.scitotenv.2019.07.354, PMID: 31369894

[ref79] Zhang D. Cai X. W. Diao L. F. Wang Y. Q. Wang J. S. An S. Q.. (2022). Changes in soil organic carbon and nitrogen pool sizes, dynamics, and biochemical stability during ~160 years natural vegetation restoration on the Loess Plateau, China. Catena 211, 106014. doi: 10.1016/j.catena.2021.106014

[ref80] Zhang J. Chen H. S. Fu Z. Y. Wang K. L. (2021). Effects of vegetation restoration on soil properties along an elevation gradient in the karst region of Southwest China. Agric. Ecosyst. Environ. 320, 107572. doi: 10.1016/j.agee.2021.107572

[ref81] Zhang C. Liu G. Xue S. Wang G. (2016). Soil bacterial community dynamics reflect changes in plant community and soil properties during the secondary succession of abandoned farmland in the Loess Plateau. Soil Biol. Biochem. 97, 40–49. doi: 10.1016/j.soilbio.2016.02.013

[ref82] Zhao H. Li X. Z. Zhang Z. M. Yang J. T. Zhao Y. Yang Z.. (2019). Effects of natural vegetative restoration on soil fungal and bacterial communities in bare patches of the southern Taihang Mountains. Ecol. Evol. 9, 10432–10441. doi: 10.1002/ece3.5564, PMID: 31624558 PMC6787810

[ref83] Zhong Y. Q. W. Yan W. M. Wang R. W. Wang W. Shangguan Z. P. (2018). Decreased occurrence of carbon cycle functions in microbial communities along with long-term secondary succession. Soil Biol. Biochem. 123, 207–217. doi: 10.1016/j.soilbio.2018.05.017

[ref84] Zhu G. Y. Shangguan Z. P. Deng L. (2021). Dynamics of water-stable aggregates associated organic carbon assessed from delta C-13 changes following temperate natural forest development in China. Soil Till. Res. 205, 104782. doi: 10.1016/j.still.2020.104782

[ref85] Zimudzi J. van der Waals J. E. Coutinho T. A. Cowan D. A. Valverde A. (2018). Temporal shifts of fungal communities in the rhizosphere and on tubers in potato fields. Fungal Biol. 122, 928–934. doi: 10.1016/j.funbio.2018.05.008, PMID: 30115327

[ref86] Zuo X. A. Wang S. K. Lv P. Zhou X. Zhao X. Y. Zhang T. H.. (2016). Plant functional diversity enhances associations of soil fungal diversity with vegetation and soil in the restoration of semiarid sandy grassland. Ecol. Evol. 6, 318–328. doi: 10.1002/ece3.1875, PMID: 26811795 PMC4716495

