# Supplementary material for: Responses of soil fungal communities and functional guilds to ~160 years of natural revegetation in the Loess Plateau of China
Source: Front Microbiol. 2022 Sep 2;13:967565. doi: 10.3389/fmicb.2022.967565 (PMC9479326; doi:10.3389/fmicb.2022.967565)
Supplement: Supplementary file 1 [file Data_Sheet_1.docx]

**Table S1** Geographical features and vegetation during various revegetation stages in the Loess Plateau of China.

| Characteristics | Revegetation stages | | | | | |
| --- | --- | --- | --- | --- | --- | --- |
|  | Farmland (S1) | Pioneer weed (S2) | Herbage (S3) | Shrub (S4) | Early forest (S5) | Climax forest (S6) |
| Longitude (E) | 109°01'52" | 109°01'52" | 109°01'44" | 109°01'00" | 109°00'56" | 109°01'47" |
| Latitude (N)  (N) | 36°00'39" | 36°00'41" | 36°00'14" | 36°00'24" | 36°00'20" | 36°01'09" |
| Altitude (m)  (m) | 1245.13 | 1238.42 | 1119.10 | 1037.98 | 1074.21 | 1201.30 |
| Aspect (°) | NE73 | NE45 | NW30 | NE20 | NE60 | NE56 |
| Slope (°) | 2–7 | 8–15 | 8–18 | 9–20 | 9–23 | 10–21 |
| Coverage (%) | 32–40 | 70–85 | 80–95 | 40–90 | 40–60 | 55–70 |
| Mean height (m) | 2.1 | 0.5 | 2.3 | 3.2 | 11.0 | 12.0 |
| Mean DBH (m) | - | - | - | - | 11.28 | 17.15 |
| Mean tree density (tree/ha) | - | - | - | - | 956 | 600 |
| Dominant species | *Zea mays L.* | *A. lavandulaefolia,*  *S. bungeana* | *T. sacchariflora* | *H. rhamnoides* | *P. davidiana* | *Q. liaotungensis* |
| Minor plant species | - | *A. gmelinii*,  *L. barystachys*,  *P. discolor*,  *V. sepium* | *A. pilosa*,  *H. altaicus*,  *C. lanceolata*,  *R. cordifolia*,  *P. discolor* | *C. lanceolata*,  *S. bungeana*,  *C. indicum*,  *P. humile* | *R. xanthina*,  *V. schensianum*,  *L. ferdinandii*,  *H. rhamnoides*,  *L. maackii*,  *H. rhamnoides* | *V. schensianum*,  *S. pubescens*,  *L. maackii*,  *L. ferdinandii*,  *S. pekinensis*,  *R. xanthina* |

**Table S2** Plant and soil properties of various revegetation stages in the Loess Plateau of China.

| Characteristics | Revegetation stages | | | | | |
| --- | --- | --- | --- | --- | --- | --- |
|  | Farmland (S1) | Pioneer weed (S2) | Herbage (S3) | Shrub (S4) | Early forest (S5) | Climax forest (S6) |
| PR | 1±0.00^d^ | 10±1^abc^ | 8±1^bc^ | 7±1^c^ | 13±2^a^ | 11±2^ab^ |
| PD | 0^d^ | 1.44±0.06^b^ | 0.90±0.13^c^ | 1.49±0.25^ab^ | 1.87±0.05^a^ | 1.70±0.08^ab^ |
| PE | 0^d^ | 0.64±0.05^b^ | 0.43±0.04^c^ | 0.82±0.07^a^ | 0.75±0.05^ab^ | 0.72±0.03^ab^ |
| LB (g m^–2^) | 25±8^c^ | 64±6^c^ | 377±77^bc^ | 432±60^bc^ | 567±32^b^ | 956±297^a^ |
| RB (g m^–2^) | 2901±188^a^ | 1588±232^b^ | 1107±141^b^ | 1485±137^b^ | 3094±410^a^ | 3356±154^a^ |
| Litter C:N ratio | 30.49±2.02^c^ | 37.10±1.21^ab^ | 36.78±1.90^ab^ | 31.50±1.04^bc^ | 32.36±2.95^bc^ | 40.37±1.36^a^ |
| Moisture (%) | 20.51±0.23^d^ | 20.90±0.14^cd^ | 20.23±0.62^d^ | 23.02±0.31^c^ | 26.17±1.47^b^ | 29.61±0.87^a^ |
| pH | 8.36±0.06^a^ | 8.35±0.03^a^ | 8.38±0.04^a^ | 8.18±0.03^bc^ | 8.28±0.05^ab^ | 8.05±0.05^c^ |
| SOC (g kg^–1^) | 9.74±0.64^cd^ | 12.03±0.56^c^ | 8.15±0.32^d^ | 11.94±1.00^c^ | 16.77±1.07^b^ | 21.76±1.26^a^ |
| WSOC (mg kg^–1^) | 46.79±6.72^c^ | 50.55±4.06^c^ | 46.58±1.54^c^ | 53.38±2.39^bc^ | 64.79±2.95^b^ | 88.95±6.63^a^ |
| TN (g kg^–1^) | 2.44±0.03^cd^ | 2.53±0.02^bc^ | 2.29±0.02^d^ | 2.68±0.11^b^ | 2.71±0.05^ab^ | 2.89±0.08^a^ |
| SON (g kg^–1^) | 1.18±0.06^bc^ | 1.33±0.04^b^ | 1.01±0.07^c^ | 1.32±0.01^b^ | 1.66±0.09^a^ | 1.86±0.09^a^ |
| NH_4_^+^-N (mg kg^–1^) | 20.62±2.28^b^ | 25.73±1.90^ab^ | 23.32±1.24^ab^ | 24.18±1.46^ab^ | 24.67±2.10^ab^ | 29.36±3.86^a^ |
| NO_3_^–^-N (mg kg^–1^) | 11.26±1.01^a^ | 3.84±0.46^b^ | 3.66±0.59^b^ | 9.93±1.06^a^ | 5.85±1.00^b^ | 5.33±1.57^b^ |
| TP (g kg^–1^) | 0.31±0.03^a^ | 0.29±0.05^a^ | 0.24±0.02^a^ | 0.21±0.03^a^ | 0.18±0.01^a^ | 0.22±0.01^a^ |
| SOC:SON | 8.27±0.21^d^ | 9.02±0.19^c^ | 8.13±0.20^d^ | 9.02±0.23^c^ | 10.12±0.27^b^ | 11.72±0.19^a^ |

Different superscript lower-case letters imply statistically significant differences at the α = 0.05 level between revegetation stages. PR: plant species richness; PD: plant species diversity; PE: plant species evenness; LB : litter biomass; RB: root biomass; SOC: soil organic carbon; WSOC: soil water-soluble organic carbon; TN: total nitrogen; SON: soil organic nitrogen; NH_4_^+^-N: ammonium nitrogen; NO_3_^–^-N: nitrate nitrogen; TP: total phosphorus.

**Table S3** Relative abundance (% of individual taxonomic groups) of the dominant fungal families (mean ± SE, n = 4) present in the soil (0–20 cm depth) microbial communities during various revegetation stages in the Loess Plateau of China.

| Family | Revegetation stages | | | | | | Source of variation |
| --- | --- | --- | --- | --- | --- | --- | --- |
|  | Farmland (S1) | Pioneer weed (S2) | Herbage (S3) | Shrub (S4) | Early forest (S5) | Climax forest (S6) |  |
| *Mortierellaceae* | 15.82±1.50^ab^ | 9.55±1.11^c^ | 16.76±2.47^ab^ | 19.35±1.56^a^ | 12.45±1.76^bc^ | 2.23±0.29^d^ | ^**^ |
| *Hygrophoraceae* | 0.00±0.00^a^ | 7.45±5.57^a^ | 1.07±0.54^a^ | 0.35±0.25^a^ | 4.98±4.73^a^ | 0.08±0.07^a^ | ^*^ |
| *Ascomycota_unclassified* | 4.13±0.53^b^ | 10.14±1.83^ab^ | 11.86±1.80^a^ | 9.49±0.69^ab^ | 6.52±2.16^ab^ | 4.71±1.81^b^ | ^*^ |
| *Clavicipitaceae* | 5.00±1.21^ab^ | 15.89±7.64^a^ | 8.49±4.01^ab^ | 6.89±1.91^ab^ | 2.59±0.34^b^ | 1.04±0.51^b^ | ^**^ |
| *Fungi_unclassified* | 4.48±0.67^b^ | 5.70±1.73^b^ | 9.83±0.99^a^ | 5.83±0.76^b^ | 4.85±0.87^b^ | 1.36±0.31^c^ | ^**^ |
| *Chaetomiaceae* | 15.69±3.00^a^ | 2.13±0.69^b^ | 2.78±0.51^b^ | 2.72±0.74^b^ | 0.71±0.15^b^ | 0.16±0.04^b^ | ^**^ |
| *Aspergillaceae* | 0.39±0.12^c^ | 1.02±0.41^c^ | 1.44±0.23^c^ | 2.50±0.80^bc^ | 8.47±3.38^a^ | 6.51±1.27^ab^ | ^*^ |
| *Nectriaceae* | 7.12±1.11^a^ | 2.19±0.30^bc^ | 3.69±0.58^b^ | 5.84±0.48^a^ | 1.52±0.18^cd^ | 0.16±0.04^d^ | ^**^ |
| *Helotiaceae* | 2.19±0.37^b^ | 0.99±0.12^b^ | 1.11±0.05^b^ | 2.10±0.39^b^ | 0.63±0.18^b^ | 13.28±3.57^a^ | ^**^ |
| *Cortinariaceae* | 0.10±0.05^b^ | 0.00±0.00^b^ | 0.01±0.01^b^ | 0.02±0.01^b^ | 0.60±0.27^b^ | 16.12±9.11^a^ | ^**^ |
| *Herpotrichiellaceae* | 0.86±0.21^d^ | 2.42±0.36^bc^ | 2.30±0.40^bcd^ | 1.42±0.28^cd^ | 3.15±0.41^b^ | 5.34±0.87^a^ | ^**^ |
| *Trichomeriaceae* | 1.40±0.27^bc^ | 1.72±0.62^bc^ | 3.06±0.43^ab^ | 4.41±1.43^a^ | 3.23±0.46^ab^ | 0.36±0.15^c^ | ^**^ |
| *Piskurozymaceae* | 4.90±1.19^a^ | 1.69±0.45^b^ | 1.50±0.22^b^ | 2.38±0.74^b^ | 2.31±0.49^b^ | 0.31±0.14^b^ | ^**^ |
| *Inocybaceae* | 0.03±0.01^b^ | 0.00±0.00^b^ | 0.00±0.00^b^ | 0.01±0.01^b^ | 10.56±6.26^a^ | 3.36±1.72^ab^ | ^**^ |
| *Sebacinaceae* | 0.05±0.02^b^ | 0.13±0.02^b^ | 0.08±0.04^b^ | 0.01±0.01^b^ | 2.92±1.15^b^ | 7.71±2.79^a^ | ^**^ |
| *Cordycipitaceae* | 0.34±0.11^b^ | 0.66±0.25^b^ | 0.50±0.09^b^ | 1.55±0.45^b^ | 0.27±0.18^b^ | 3.07±0.98^a^ | ^*^ |
| *Tricholomataceae* | 0.04±0.03^b^ | 0.01±0.01^b^ | 0.01±0.01^b^ | 0.26±0.11^b^ | 0.12±0.03^b^ | 7.42±4.74^a^ | ^*^ |
| *Trichocomaceae* | 0.23±0.07^b^ | 0.73±0.63^b^ | 0.07±0.02^b^ | 0.33±0.10^b^ | 3.88±1.32^a^ | 1.30±0.42^b^ | ^*^ |
| *Didymellaceae* | 3.93±0.72^a^ | 0.64±0.16^b^ | 0.68±0.22^b^ | 0.42±0.08^b^ | 0.91±0.67^b^ | 0.01±0.00^b^ | ^**^ |
| *Lasiosphaeriaceae* | 2.86±0.21^a^ | 0.35±0.10^c^ | 0.45±0.13^c^ | 1.66±0.37^b^ | 0.06±0.04^c^ | 0.02±0.01^c^ | ^**^ |
| *Russulaceae* | 0.03±0.01^b^ | 0.00±0.00^b^ | 0.01±0.01^b^ | 0.00±0.00^b^ | 0.01±0.00^b^ | 5.47±1.80^a^ | ^**^ |
| *Thelephoraceae* | 0.02±0.01^c^ | 0.01±0.01^c^ | 0.08±0.05^c^ | 0.09±0.03^c^ | 2.75±0.72^a^ | 1.72±0.36^b^ | ^**^ |
| *Sordariales_unclassified* | 0.50±0.08^b^ | 0.38±0.07^b^ | 0.41±0.14^b^ | 0.90±0.55^b^ | 0.25±0.12^b^ | 2.03±0.66^a^ | n.s. |
| *Sporormiaceae* | 2.63±0.87^a^ | 0.75±0.19^b^ | 0.35±0.10^b^ | 0.42±0.08^b^ | 0.07±0.02^b^ | 0.02±0.01^b^ | ^**^ |
| *Myxotrichaceae* | 0.02±0.01^b^ | 0.06±0.05^b^ | 0.04±0.04^b^ | 0.04±0.02^b^ | 0.93±0.76^ab^ | 2.17±0.85^a^ | ^*^ |
| *Trichosporonaceae* | 0.83±0.08^a^ | 0.38±0.10^bc^ | 0.42±0.03^bc^ | 0.73±0.16^ab^ | 0.20±0.06^c^ | 0.16±0.04^c^ | ^**^ |
| *Stachybotryaceae* | 1.07±0.24^a^ | 0.15±0.04^bc^ | 0.37±0.03^b^ | 0.31±0.09^bc^ | 0.07±0.02^bc^ | 0.00±0.00^c^ | ^**^ |
| *Myrmecridiaceae* | 1.04±0.25^a^ | 0.06±0.01^b^ | 0.09±0.03^b^ | 0.14±0.06^b^ | 0.01±0.01^b^ | 0.00±0.00^b^ | ^**^ |

^*^ P < 0.05; ^**^ P < 0.01; n.s.: not significant. Different superscript lower-case letters imply statistically significant differences at the α = 0.05 level between revegetation stages.

**Table S4** Effects of revegetation stages on soil fungal communities at the OTU-level with ANOISM analysis, using Bray-Curtis distances with 999 permutations.

| Method  ANOSIM | Statistic R  ANOSIM | P value | Permutation_number |
| --- | --- | --- | --- |
| ANOSIM | 0.9257 | 0.001 | 999 |

**Table S5** Effects of revegetation stages and environmental factors on soil fungal communities at the OTU-level as assessed by permutational multivariate analysis of variance (PERMANOVA), using Bray-Curtis distances with 999 permutations.

| Characteristics | SumsOfSqs | MeanSqs | F.Model | R^2^ | P. value | P. adjust |
| --- | --- | --- | --- | --- | --- | --- |
| Revegetation stages | 4.06814 | 0.81363 | 4.60414 | 0.56120 | 0.001 | 0.00142 |
| PR | 1.00521 | 1.00521 | 3.54185 | 0.13867 | 0.001 | 0.00142 |
| PD | 1.07852 | 1.07852 | 3.84529 | 0.14878 | 0.001 | 0.00142 |
| LB | 1.06545 | 1.06545 | 3.79066 | 0.14698 | 0.001 | 0.00142 |
| RB | 0.91470 | 0.91470 | 3.17689 | 0.12618 | 0.001 | 0.00142 |
| Moisture | 1.51732 | 1.51732 | 5.82391 | 0.20931 | 0.001 | 0.00142 |
| pH | 1.05578 | 1.05578 | 3.75041 | 0.14564 | 0.001 | 0.00142 |
| SOC | 1.52091 | 1.52091 | 5.84137 | 0.20981 | 0.001 | 0.00142 |
| WSOC | 1.42075 | 1.42075 | 5.36290 | 0.19599 | 0.001 | 0.00142 |
| TN | 1.10471 | 1.10471 | 3.95548 | 0.15239 | 0.001 | 0.00142 |
| SON | 1.31660 | 1.31660 | 4.88251 | 0.18162 | 0.001 | 0.00142 |
| SOC:SON | 1.56628 | 1.56628 | 6.06364 | 0.21607 | 0.001 | 0.00142 |
| PE | 0.92210 | 0.92210 | 3.20631 | 0.12720 | 0.002 | 0.00262 |
| NO_3_^–^-N | 0.71043 | 0.71043 | 2.39035 | 0.09800 | 0.007 | 0.00850 |
| Litter C:N ratio | 0.65467 | 0.65467 | 2.18409 | 0.09031 | 0.011 | 0.01247 |
| TP | 0.61405 | 0.61405 | 2.03604 | 0.08471 | 0.018 | 0.01913 |
| NH_4_^+^-N | 0.56181 | 0.56181 | 1.84829 | 0.07750 | 0.034 | 0.0340 |

SumsOfSqs: Sums of squares; MeanSqs: Mean squares; F.Model: F value; See Table S2 for abbreviations.

**Table S6** Effects of revegetation stages and environmental factors on soil fungal communities at the phylum-level as assessed by permutational multivariate analysis of variance (PERMANOVA), using Bray-Curtis distances with 999 permutations.

| Characteristics | SumsOfSqs | MeanSqs | F.Model | R^2^ | P. value | P. adjust |
| --- | --- | --- | --- | --- | --- | --- |
| Revegetation stages | 0.52486 | 0.10497 | 8.94224 | 0.71297 | 0.001 | 0.00340 |
| Moisture | 0.39468 | 0.39468 | 25.42767 | 0.53614 | 0.001 | 0.00340 |
| SOC | 0.36386 | 0.36386 | 21.50110 | 0.49427 | 0.001 | 0.00340 |
| SON | 0.32215 | 0.32215 | 17.11858 | 0.43761 | 0.001 | 0.00340 |
| SOC:SON | 0.35577 | 0.35577 | 20.57581 | 0.48327 | 0.001 | 0.00340 |
| WSOC | 0.27902 | 0.27902 | 13.42781 | 0.37902 | 0.002 | 0.00567 |
| LB | 0.27458 | 0.27458 | 13.08682 | 0.37298 | 0.003 | 0.00729 |
| TN | 0.21478 | 0.21478 | 9.06284 | 0.29176 | 0.004 | 0.00850 |
| PR | 0.20127 | 0.20127 | 8.27796 | 0.27340 | 0.005 | 0.00944 |
| Litter C:N ratio | 0.15330 | 0.15330 | 5.78648 | 0.20825 | 0.011 | 0.01870 |
| PD | 0.15568 | 0.15568 | 5.90009 | 0.21147 | 0.015 | 0.02267 |
| RB | 0.15539 | 0.15539 | 5.88602 | 0.21107 | 0.016 | 0.02267 |
| pH | 0.14929 | 0.14929 | 5.59660 | 0.20280 | 0.020 | 0.02615 |
| NO_3_^–^-N | 0.11021 | 0.11021 | 3.87356 | 0.14971 | 0.045 | 0.05464 |
| PE | 0.09049 | 0.09049 | 3.08310 | 0.12292 | 0.079 | 0.08953 |
| NH_4_^+^-N | 0.07749 | 0.07749 | 2.58817 | 0.10526 | 0.096 | 0.10200 |
| TP | 0.00665 | 0.00665 | 0.20044 | 0.00903 | 0.792 | 0.79200 |

See Tables S2 and S5 for abbreviations.

**Table S7** Effects of revegetation stages and environmental factors on soil fungal communities at the class-level as assessed by permutational multivariate analysis of variance (PERMANOVA), using Bray-Curtis distances with 999 permutations.

| Characteristics | SumsOfSqs | MeanSqs | F.Model | R^2^ | P. value | P. adjust |
| --- | --- | --- | --- | --- | --- | --- |
| Revegetation stages | 1.47711 | 0.29542 | 11.21502 | 0.75700 | 0.001 | 0.00189 |
| PR | 0.58509 | 0.58509 | 9.42192 | 0.29985 | 0.001 | 0.00189 |
| LB | 0.64837 | 0.64837 | 10.94813 | 0.33228 | 0.001 | 0.00189 |
| Moisture | 0.97029 | 0.97029 | 21.76046 | 0.49726 | 0.001 | 0.00189 |
| SOC | 0.96554 | 0.96554 | 21.54983 | 0.49483 | 0.001 | 0.00189 |
| WSOC | 0.84387 | 0.84387 | 16.76498 | 0.43248 | 0.001 | 0.00189 |
| TN | 0.62808 | 0.62808 | 10.44294 | 0.32189 | 0.001 | 0.00189 |
| SON | 0.84078 | 0.84078 | 16.65698 | 0.43089 | 0.001 | 0.00189 |
| SOC:SON | 0.97207 | 0.97207 | 21.83991 | 0.49817 | 0.001 | 0.00189 |
| PD | 0.56746 | 0.56746 | 9.02153 | 0.29082 | 0.002 | 0.00309 |
| Litter C:N ratio | 0.41530 | 0.41530 | 5.94839 | 0.21283 | 0.002 | 0.00309 |
| pH | 0.43089 | 0.43089 | 6.23508 | 0.22083 | 0.003 | 0.00425 |
| PE | 0.39552 | 0.39552 | 5.59322 | 0.20270 | 0.009 | 0.01177 |
| NH_4_^+^-N | 0.32590 | 0.32590 | 4.41118 | 0.16702 | 0.012 | 0.01457 |
| RB | 0.35249 | 0.35249 | 4.85040 | 0.18065 | 0.013 | 0.01473 |
| NO_3_^–^-N | 0.28924 | 0.28924 | 3.82859 | 0.14823 | 0.022 | 0.02338 |
| TP | 0.18492 | 0.18492 | 2.30320 | 0.09477 | 0.109 | 0.10900 |

See Tables S2 and S5 for abbreviations.
